# CRISPR-assisted rational flux-tuning and arrayed CRISPRi screening of an l-proline exporter for l-proline hyperproduction

**DOI:** 10.1038/s41467-022-28501-7

**Published:** 2022-02-16

**Authors:** Jiao Liu, Moshi Liu, Tuo Shi, Guannan Sun, Ning Gao, Xiaojia Zhao, Xuan Guo, Xiaomeng Ni, Qianqian Yuan, Jinhui Feng, Zhemin Liu, Yanmei Guo, Jiuzhou Chen, Yu Wang, Ping Zheng, Jibin Sun

**Affiliations:** 1 Key Laboratory of Systems Microbial Biotechnology, Tianjin Institute of Industrial Biotechnology, Chinese Academy of Sciences, Tianjin, 300308 China; 2 National Technology Innovation Center of Synthetic Biology, Tianjin, 300308 China; 3 University of Chinese Academy of Sciences, Beijing, 100049 China

**Keywords:** Metabolic engineering, Applied microbiology, Transporters

## Abstract

Development of hyperproducing strains is important for biomanufacturing of biochemicals and biofuels but requires extensive efforts to engineer cellular metabolism and discover functional components. Herein, we optimize and use the CRISPR-assisted editing and CRISPRi screening methods to convert a wild-type *Corynebacterium glutamicum* to a hyperproducer of l-proline, an amino acid with medicine, feed, and food applications. To facilitate l-proline production, feedback-deregulated variants of key biosynthetic enzyme γ-glutamyl kinase are screened using CRISPR-assisted single-stranded DNA recombineering. To increase the carbon flux towards l-proline biosynthesis, flux-control genes predicted by in silico analysis are fine-tuned using tailored promoter libraries. Finally, an arrayed CRISPRi library targeting all 397 transporters is constructed to discover an l-proline exporter Cgl2622. The final plasmid-, antibiotic-, and inducer-free strain produces l-proline at the level of 142.4 g/L, 2.90 g/L/h, and 0.31 g/g. The CRISPR-assisted strain development strategy can be used for engineering industrial-strength strains for efficient biomanufacturing.

## Abstract (web summary)

*Corynebacterium glutamicum* is a major workhorse in industrial biomanufacturing of amino acids. Here, the authors employ CRISPR-assisted rational flux-tuning and CRISPRi screening of a L-proline exporter to covert a wild-type C. glutamicum to a hyperproducer of L-proline.

## Introduction

Industrial biomanufacturing that converts biomass-derived carbon sources into target chemicals holds promise for addressing global concerns over limited fossil resources and environmental problems. Microbial strains are considered as the key biocatalysts of biomanufacturing^1^. However, it is still challenging and time-consuming to develop microbial strains that meet the requirements of industrialization and commercialization, such as high production level (titer, yield, and productivity), free of plasmid, inducer, and antibiotic, and stable performance in scale-up fermentation^2–4^. Random mutagenesis and screening strategies are widely used in the initial stage of strain development, especially in the breeding of amino acid producers^3,5,6^. Stepwise rational metabolic engineering at a systems level can further improve strain performance but also requires efficient genome editing tools and components with specific functions like catalysis, regulation, and transport^7–9^.

The CRISPR (Clustered regularly interspaced short palindromic repeats) system has recently been explored as a leading-edge tool for microbial genome editing. Besides genome editing applications such as gene deletion and insertion^10^, the CRISPR system offers enormous potential for genotype-phenotype mapping at a genome scale using arrayed or pooled guide RNA (gRNA) libraries^11^. Genome-wide pooled CRISPR interference (CRISPRi) screening has been performed in several microorganisms including *Saccharomyces cerevisiae*^12^, *Escherichia coli*^13^, and *Synechocystis*^14^ for identifying crucial genes for viability and tolerance. Conversely, arrayed CRISPRi libraries for microorganisms have been rarely reported, possibly due to the higher cost and demand for library construction and screening methods. Only arrayed CRISPRi libraries targeting ~300 essential genes were constructed for *Bacillus subtilis*^15^ and *Streptococcus pneumoniae*^16^. Its potential in mining functional components for industrial strain development has not been fully exploited.

*Corynebacterium glutamicum* is a major workhorse in industrial biomanufacturing^17^. It is now used for the industrial production of over six million tons of amino acids (such as l-lysine and l-glutamate) per year. Additionally, this microorganism has been engineered to produce many more compounds ranging from alcohols, organic acids, and plant secondary metabolites^18^. l-Proline, the only proteinogenic amino acid with a secondary amine, is a high-value amino acid with applications in the medicine and food industry and has large potential for use as a feed additive^19,20^. Compared with the fermentative production of amino acids like l-lysine and l-glutamate with a high yield of ~0.70 g product/g glucose^21,22^, the l-proline production has a relatively low yield of ~0.20 g/g^23^ (Supplementary Table 1). Plasmid-based inducible expression systems requiring antibiotics and expensive chemical inducers are still used in l-proline producing strains^23,24^, which preclude their use in industrial-scale fermentation. In the process of transforming *C. glutamicum* into efficient producers of molecules of interest, transport engineering has played a prominent role besides metabolic engineering^25,26^. Traditional methods for discovering carrier proteins for molecule transport include phenotype screening of random mutation or genomic DNA libraries^27,28^, transcriptome analysis^29^, and sequence similarity search^30^. Because of the limitations of these methods in comprehensiveness and universality, exporters for many compounds, including l-proline, are yet to be discovered^25,26^.

In this study, to de novo develop an industrial-strength l-proline producing strain, we first optimize the CRISPR system in *C. glutamicum* for decreasing the cytotoxicity and increasing editing efficiency. CRISPR-assisted chromosomal editing and arrayed CRISPRi screening are then employed for engineering crucial enzymes, fine-tuning metabolic fluxes, and discovering l-proline exporters. Fed-batch fermentations of the final plasmid-, antibiotic-, and inducer-free strain result in the highly efficient production of l-proline. This study demonstrates how CRISPR facilitates de novo development of high-performance strains by enabling efficient chromosomal engineering and the discovery of functional components. The arrayed CRISPRi library for all *C. glutamicum* transporters will be useful for investigating the transport of other molecules of interest.

## Results

### Optimization of CRISPR/Cas9-assisted genome editing methods for *C. glutamicum*

Efficient methods for genetic manipulation are required for strain development. Genome editing tools based on both CRISPR/Cas9^31–34^ and CRISPR/Cas12a^35–38^ have been developed for *C. glutamicum*. Comparing these two systems for application in GC-rich *C. glutamicum* (53.8% GC-content for type strain ATCC 13032), the CRISPR/Cas9 system recognizing G-rich protospacer adjacent motif (PAM) sequences should have a wider genome targeting scope than the CRISPR/Cas12a system recognizing T-rich PAMs^39^. Besides, the CRISPR/Cas9 system can distinguish a single nucleotide change in the seed sequence, which is preferred for precise editing^40^. However, the CRISPR/Cas9 system is cytotoxic to *C. glutamicum* and previous methods usually suffer from few transformants and low accuracy, especially for manipulation of large DNA fragments^32,34^.

**Fig. 1:**
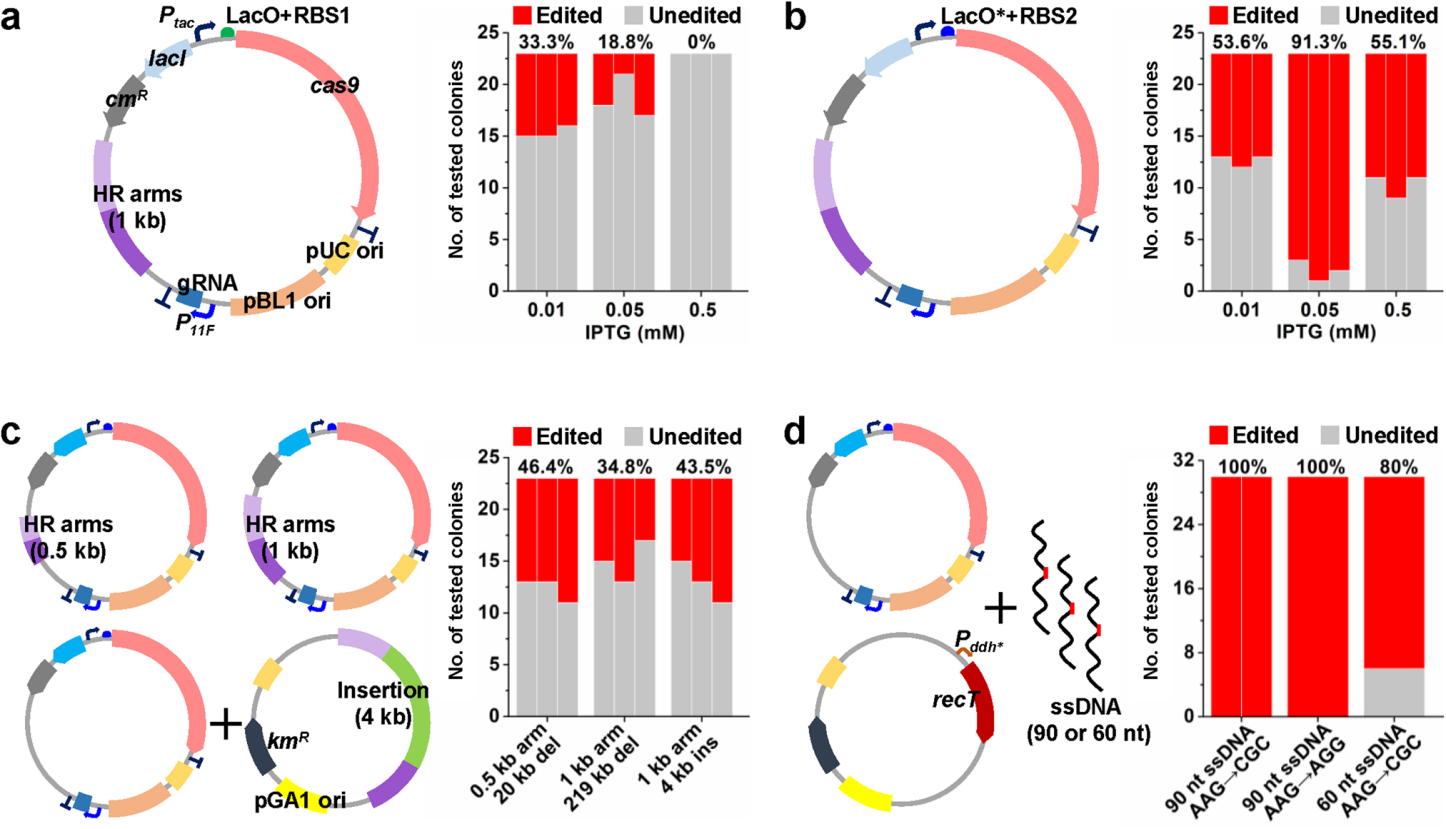
Gene deletion, insertion, and ssDNA recombineering in *C. glutamicum* using the optimized CRISPR/Cas9 system. **a** Deletion of a 1.7 kb DNA fragment (*cgl0620*-*cgl0622*) using the all-in-one plasmid. Different concentrations of IPTG (0.01, 0.05, and 0.5 mM) were used for counter-selection. **b** Deletion of the same 1.7 kb DNA fragment (*cgl0620*-*cgl0622*) using the optimized all-in-one plasmid with a modified *lac* operator that binds LacI very tightly (LacO^*^) and a weak RBS (RBS2). Different concentrations of IPTG (0.01, 0.05, and 0.5 mM) were used for counter-selection. **c** Deletion and insertion of large DNA fragments. Three tests, deletion of a 20 kb DNA fragment (part of prophage CGP3) with 0.5 kb HR arms, deletion of a 219 kb DNA fragment (complete prophage CGP3) with 1.0 kb HR arms, and insertion of a 4 kb DNA fragment (an artificial *proBAC* operon inserted to *putA*) with 1.0 kb HR arms were conducted. For gene insertion, the inserted DNA fragment and HR arms were provided using a second plasmid. IPTG (0.05 mM) was used for counter-selection. For **a**, **b**, and **c**, twenty-three colonies were randomly selected and verified by PCR for each test. Results of three independent replicates are shown. Red and grey fractions of bars represent edited and unedited colonies, respectively. The average editing efficiency (%) from three independent replicates is shown above the bars. **d** Introduction of triple and single nucleotide changes using CRISPR/Cas9-assisted ssDNA recombineering. Three tests, triple nucleotide changes with 90 nt ssDNA (two independent replicates), single nucleotide change with 90 nt ssDNA, and triple nucleotide changes with 60 nt ssDNA, were conducted to introduce *rpsL*^K43R^ mutation that produced streptomycin resistance phenotype. IPTG (0.05 mM) was used for counter-selection. Thirty colonies were randomly selected and verified by streptomycin resistance phenotype test. Red and grey fractions of bars represent edited and unedited colonies, respectively. The editing efficiency (%) is shown above the bars. Genetic elements on the plasmids: *P*_*tac*_, IPTG-inducible *tac* promoter; *P*_*11F*_, 11F constitutive promoter^32^; *P*_ddh_^*^, a variant of the promoter of *ddh* gene (Supplementary Data 6); LacO, wild-type *lac* operator; LacO^*^, a modified *lac* operator that binds LacI tightly^42^; RBS1, a strong RBS AAAGGAGTTGAGA; RBS2, a weak RBS AAAGGCACCCGAT; pUC ori, pUC origin of replication; pBL1 ori, pBL1 origin of replication; pGA1 ori, pGA1 origin of replication; *cas9*, *S. pyogenes* Cas9 gene; gRNA, guide RNA expression cassette; HR arms, homologous recombination arms; *cm*^R^, chloramphenicol resistance gene; *km*^R^, kanamycin resistance gene; *recT*, gene encoding the recombinase from the Rac prophage of *E. coli*; Insertion, the 4 kb DNA fragment for insertion test. Images for DNA gel electrophoresis and streptomycin resistance phenotype test are provided in Supplementary Figs. 1 and 3. Source data underlying Fig. 1 are provided as a Source Data file.

The double-stranded DNA break (DSB) generated by the CRISPR/Cas9 can be a two-edged sword, which is lethal to most bacterial cells but can be used for counter-selection of edited cells^41^. Therefore, the unwanted and untimely leaky expression of Cas9 proteins should be minimized. To optimize the CRISPR/Cas9 system for *C. glutamicum*, we first constructed a temperature-sensitive plasmid that expressed Cas9 and gRNA and harbored homologous recombination (HR) arms. With a very low dosage of isopropyl-β-d-thiogalactopyranoside (IPTG) (0.01 mM) to induce Cas9-facilitated counter-selection, a few dozen transformants and a moderate editing efficiency of 33.3% were obtained for deletion of a 1.7 kb DNA fragment (*cgl0620*-*cgl0622*) with two 1.0 kb HR arms. Interestingly, with the increase in IPTG dosage, the transformant number and editing efficiency gradually decreased (Fig. 1a and Supplementary Fig. 1), again demonstrating the importance of controllable Cas9 expression in genome editing.

Next, the expression of Cas9 was regulated by using a perfectly symmetric *lac* operator (LacO) that binds the *lac* repressor (LacI) very tightly^42^ and a weaker ribosome binding site (RBS). By using a green fluorescent protein (GFP) as a reporter, it was found that these modifications almost eliminated leaky expression. Gradually enhanced gene expression was obtained by increasing the IPTG dosage (Supplementary Fig. 2a). When used for *cas9* expression, these modifications on LacO and RBS also reduced the cytotoxicity of *cas9* expressing plasmid and produced more transformants (Supplementary Fig. 2b). Then, the optimized system was tested for Cas9-mediated gene deletion. The editing efficiency was largely improved up to 91.3% with 0.05 mM IPTG dosage (Fig. 1b and Supplementary Fig. 1). Hundreds of transformants could be obtained because of the strict control of Cas9 expression. More tests were then conducted to evaluate the optimized system. When shorter HR arms (0.5 kb) were used for deletion of a longer DNA fragment (20 kb fragment in the prophage CGP3), an editing efficiency of 46.4% was obtained, which was over the previously reported efficiency of 26.9% for the same test^34^. The editing efficiency remained 34.8% for deletion of a 219 kb DNA fragment (complete prophage CGP3), which has not been achieved by previous methods^34^. For insertion of a 4 kb DNA fragment (an artificial *proBAC* operon), an efficiency of 43.5% was obtained (Fig. 1c and Supplementary Fig. 3).

With exogenous recombinases, recombination with single-stranded DNA (ssDNA) can be more efficient than recombination with plasmid-borne double-stranded DNA (dsDNA)^32,43^. To test genome editing with ssDNAs as templates, the RecT recombinase from the Rac prophage of *E. coli* was expressed using a low-copy and easily curable plasmid under the control of a strong constitutive promoter *P*_ddh_^*^. By providing 90 nt ssDNA templates targeting the *rpsL*^K43R^ mutation, triple or single nucleotide changes were efficiently introduced to the target site with 100% efficiencies and over 1000 transformants for each test. The editing efficiency remained 80% when the length of ssDNA was reduced to 60 nt (Fig. 1d and Supplementary Fig. 3). Compared with the CRISPR/Cas9 systems previously developed for *C. glutamicum*^31–34^, the present system shows overall improved editing efficiency. A routine procedure for genome editing and plasmid curing takes as short as four days (Supplementary Fig. 4). The sizable quantity of transformants also make this system easy to operate.

### Screening γ-glutamyl kinase variants to facilitate l-proline production

**Fig. 2:**
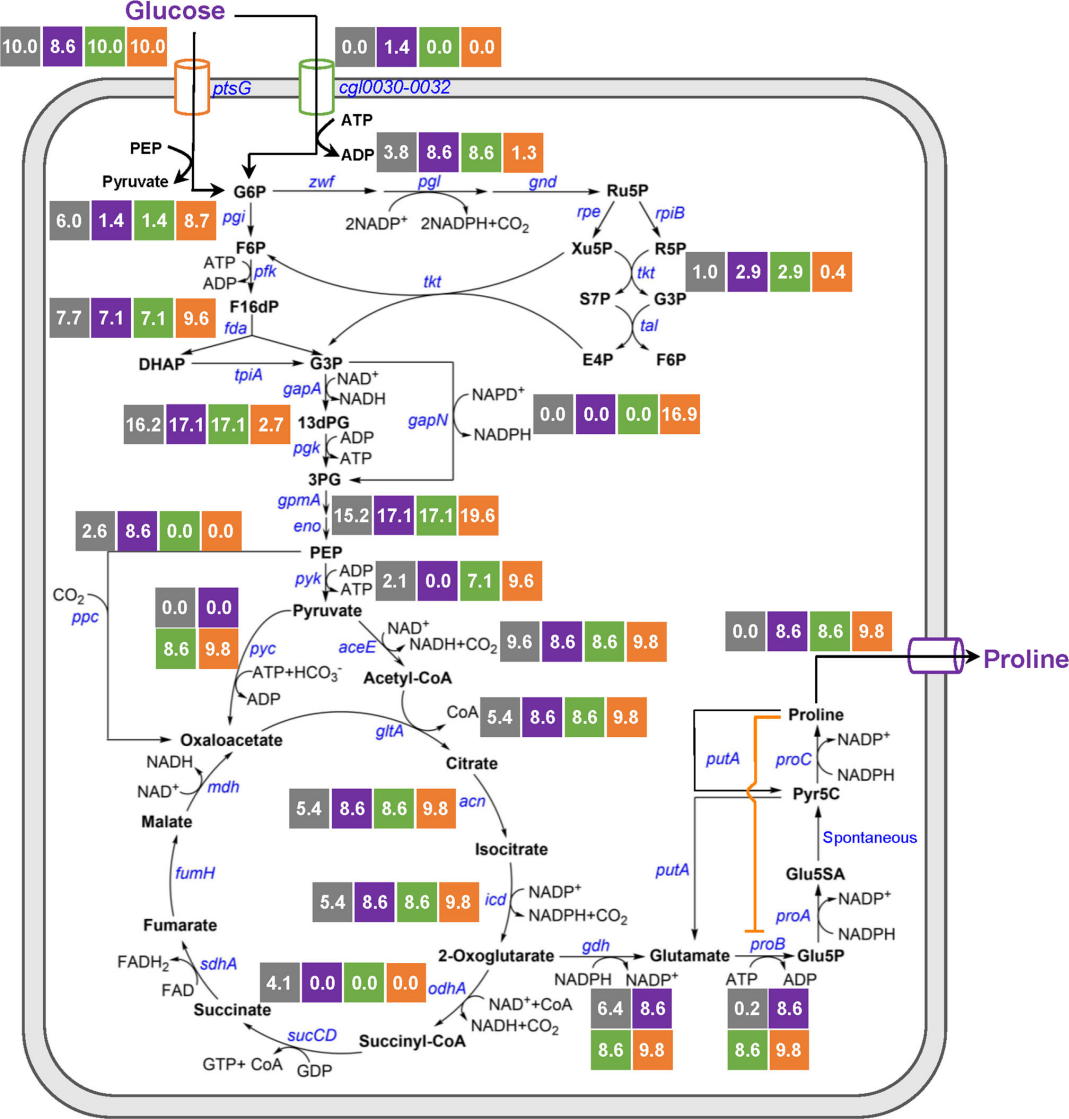
In silico simulation of the l-proline biosynthetic pathway from glucose. Glucose input was set at 10 mmol/gCDW·h. The numbers in squares represent metabolic fluxes of reactions. Grey squares represent the metabolic fluxes for maximum biomass formation. Purple squares represent the metabolic fluxes for maximum l-proline biosynthesis using the *ppc*-based C4 anaplerotic pathway. Green squares represent the metabolic fluxes for maximum l-proline biosynthesis using the *pyc*-based C4 anaplerotic pathway. Orange squares represent the metabolic fluxes for maximum l-proline biosynthesis using the *gapN*-based G3P oxidation pathway and *pyc*-based C4 anaplerotic pathway. The feedback inhibition of ProB by l-proline is indicated with orange line. Source data for in silico simulation are provided in Supplementary Data 3–5.

**Fig. 3:**
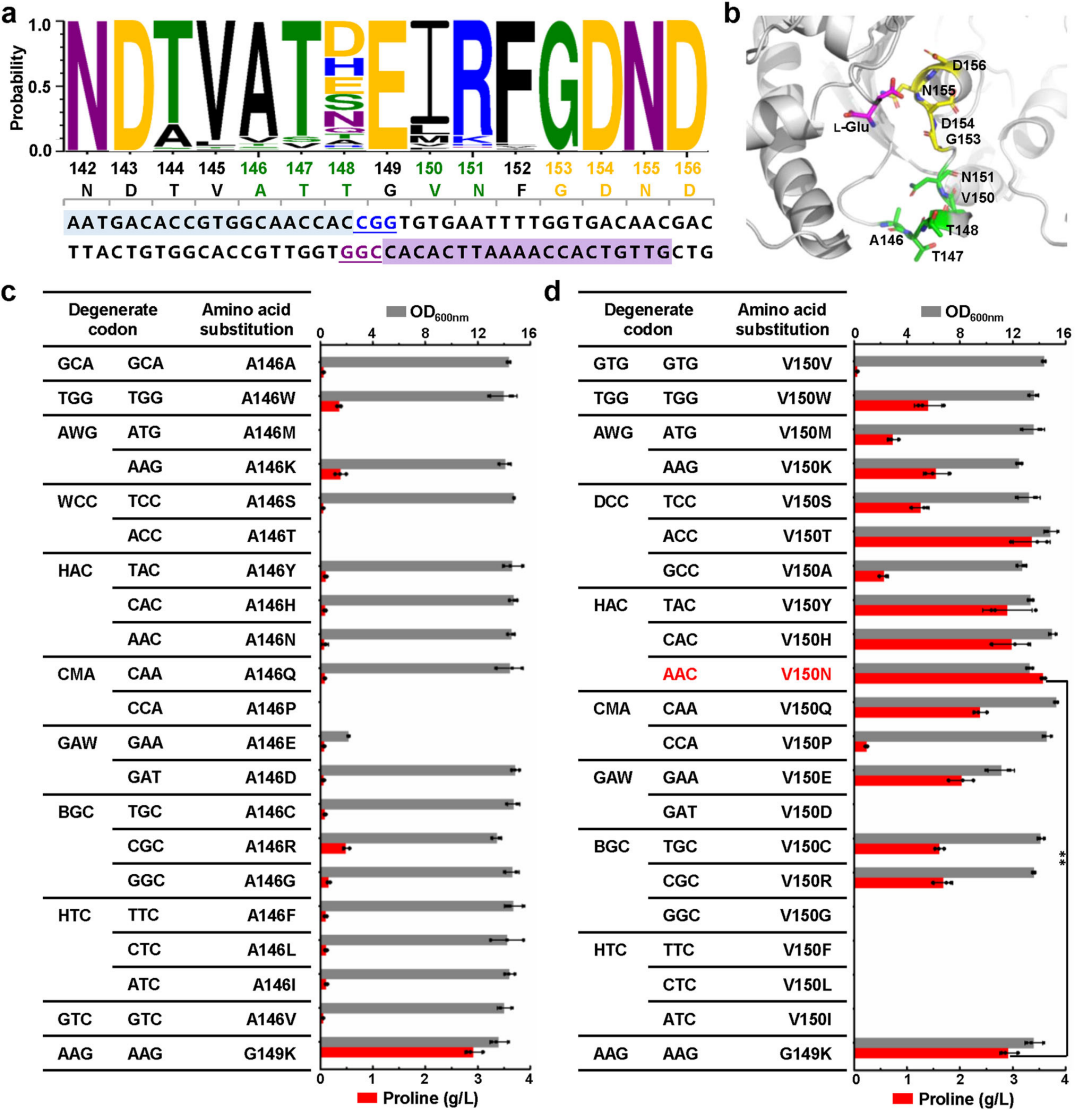
Codon saturation mutagenesis of γ-glutamyl kinase of *C. glutamicum* (CgProB) for improved l-proline production via CRISPR/Cas9-assisted ssDNA recombineering. **a** Conservation analysis of γ-glutamyl kinases. The conserved region involved in l-proline binding is indicated in yellow. The A146, T147, T148, V150, and N151 residues mutated in this study are highlighted in green. The two gRNAs used and their corresponding PAM sequences (underlined) are highlighted in blue and purple, respectively. γ-Glutamyl kinases sequences used for conservation analysis are provided in Supplementary Data 1. **b** The model structure of CgProB. The model structure was constructed with the crystal structure of γ-glutamyl kinase from *E. coli* (PDB ID: 2J5T)^80^ as a template (93% coverage and 38% sequence identity with CgProB) using Discovery Studio 2018 software. The backbone of CgProB is shown in a ribbon model on which several key residues are shown in a stick model. The substrate l-glutamate is indicated in magenta. The conserved amino acid residues G153, D154, N155, and D156 are indicated in yellow. The engineered amino acids residues A146, T147, T148, V150, and N151 are indicated in green. The full structure is shown in Supplementary Fig. 5. **c** Growth and l-proline production by *C. glutamicum* strains with amino acid substitution at codon 146 of CgProB. The strain harboring reported CgProB^G149K^ mutation^35^ was used as a control. l-Proline production was conducted in 96-deep-well plates. Data are presented as mean values +/− SD (*n* = 3 independent experiments). **d**, Growth and l-proline production by *C. glutamicum* strains with amino acid substitution at codon 150 of CgProB. The strain harboring reported CgProB^G149K^ mutation^35^ was used as a control. The V150N mutation used in strain PRO-1 is indicated in red. l-Proline production was conducted in 96-deep-well plates. Data are presented as mean values +/− SD (*n* = 3 independent experiments). **P = 0.0024, Student’s two-tailed *t*-test. Source data underlying Fig. 3c, d are provided as a Source Data file.

With the optimized genome editing method, we conducted metabolic engineering of *C. glutamicum* ATCC 13032 for l-proline production. l-Proline is synthesized from the tricarboxylic acid cycle (TCA cycle) intermediate 2-oxoglutarate by l-glutamate dehydrogenase (Gdh), γ-glutamyl kinase (ProB), γ-glutamyl phosphate reductase (ProA), spontaneous cyclization, and pyrroline-5-carboxylate reductase (ProC) in *C. glutamicum*. l-Proline biosynthesis is tightly regulated by feedback inhibition of ProB by the end-product l-proline (Fig. 2)^19^. Therefore, releasing the feedback inhibition of ProB by l-proline is the initial step for the development of l-proline producers^44^. The ~100% single nucleotide editing efficiency of CRISPR/Cas9-assisted ssDNA recombineering makes it possible to perform codon saturation mutagenesis in *C. glutamicum* chromosome without introducing adjacent synonymous mutations, which do not alter the encoded protein but can influence gene expression^45^. To identify the target amino acid residues for mutagenesis, 946 ProB sequences from UniProt database were aligned and analyzed (Fig. 3a and Supplementary Data 1). It has been reported that the substrate l-glutamate and the feedback inhibitor l-proline bind at overlapping sites^46^. The predicted binding sites of ProB from *C. glutamicum* ATCC 13032 (CgProB) are D154 and N155^35^, which are conserved in ProB enzymes from different sources. Interestingly, the adjacent region is less conserved, especially positions 148 and 150. The sequence alignment suggests that T148, G149, V150, and N151 of CgProB are all low-probability amino acid residues for these four sites (probability of 4.55, 0.95, 3.07, and 1.37%, respectively) (Fig. 3a). The 3D protein structure modelling of CgProB indicates that the less conserved region forms a flexible loop and maps away from the binding sites (Fig. 3b and Supplementary Fig. 5a). Analysis of the receptor-ligand interactions suggests that these amino acid residuals unlikely interact with the substrate l-glutamate directly (Supplementary Fig. 5b). However, previous studies on ProB of *E. coli* show that this flexible loop modulates l-proline inhibition^47^. Therefore, we hypothesize that mutations in this region may release the feedback inhibition of CgProB by l-proline without affecting the substrate l-glutamate binding. Previous research on G149 mutagenesis provides support for our hypothesis^35^. Therefore, codon saturation mutagenesis at A146, T147, T148, V150, and N151 were performed for CgProB with CRISPR/Cas9-assisted ssDNA recombineering.

To reduce the library size and avoid rare codons, 8–9 ssDNAs containing degenerate bases were designed and synthesized to cover all the 19 amino acid substitutions (Fig. 3c, d). Sixty transformants were randomly picked and tested for editing at each site. Site-directed mutagenesis targeting A146 and V150 produced several mutants with extracellular l-proline accumulation using glucose as a carbon source. Sequencing of *proB* in these mutants showed that sixteen different amino acid substitutions were obtained for A146 mutation and fourteen different amino acid substitutions were obtained for V150 mutation. All the sequenced *proB* variants had one to three nucleotide changes, demonstrating a 100% editing efficiency. Compared with A146 substitutions leading to low-level l-proline production (A146W, A146K, and A146R) (Fig. 3c), several V150 substitutions facilitated up to 3.6 g/L l-proline production (V150N), which was even 24.1% higher than that produced by the strain harboring previously reported G149K substitution (2.9 g/L) (Fig. 3d). The l-proline producing mutants overall showed decreased biomass compared with the wild-type strain, suggesting a redirection of metabolic flux from the production of biomass to the target metabolite. The strain expressing the *proB*^V150N^ mutant was designated as strain PRO-1.

### Metabolic flux analysis for optimal l-proline biosynthesis

Next, to predict the flux-control reactions leading to the optimal l-proline biosynthesis, flux balance analysis (FBA) was performed using a genome-scale metabolic model of *C. glutamicum*, *i*CW773^24^. FBA is a mathematical approach for analyzing the fluxes of metabolites through a metabolic network and has been widely used to predict the growth rate of an organism or the theoretical yield of producing a metabolite of interest^48^. By comparing the metabolic flux distribution between maximum biomass formation and maximum l-proline production, the flux-control reactions can be predicted. The in silico simulation suggests that when the substrate glucose is used for maximum biomass formation, the glycolysis and TCA cycle are active and few metabolic fluxes are channeled to l-proline biosynthesis. When maximum l-proline biosynthesis is the objective of simulation, 2-oxoglutarate flux is drained from the TCA cycle to enter the l-proline biosynthetic pathway, resulting in a theoretical l-proline yield from glucose of 0.86 mol/mol (0.55 g/g) (Fig. 2 and Supplementary Data 2 and 3). Since the TCA cycle is highly active in *C. glutamicum*^49^, a strong l-proline biosynthetic pathway (Gdh, ProB, ProA, and ProC) is expected to channel 2-oxoglutarate flux from the TCA cycle^23^.

According to the simulation, an efficient C4 anaplerotic pathway is needed for the optimal l-proline biosynthesis. The phosphoenolpyruvate carboxylase (Ppc)-based C4 anaplerotic pathway converts phosphoenolpyruvate (PEP) and CO_2_ to oxaloacetate^50^. To save PEP for oxaloacetate biosynthesis, glucose needs to be partially transported into cells via the nonphosphotransferase system (non-PTS) (Fig. 2), which is inefficient in *C. glutamicum*^51^. The pyruvate carboxylase (Pyc)-based C4 anaplerotic pathway offers a promising alternative^50^. First, Pyc converts pyruvate and CO_2_ to oxaloacetate. As a result, sufficient PEP can be used as the phosphoryl donor for phosphotransferase system (PTS)-mediated glucose uptake, which allows complete glucose uptake via the efficient PTS without the involvement of inefficient non-PTS. Second, the simulation suggests that using the Pyc-based C4 anaplerotic pathway leads to excess ATP production from glucose (2.04 excess ATP mol/mol glucose), which can support cell growth. In the simulation, the excess ATP is consumed by meaningless cyclic reactions, e.g., ATP-consuming l-cysteine transport reactions shown in Supplementary Data 4.

Since significant amount of NADPH is needed for l-proline biosynthesis, almost half carbon flux is channeled into the oxidative pentose phosphate pathway (PPP) for NADPH regeneration at the cost of CO_2_ release, which consequently lowers the l-proline yield. The nonphosphorylating NADP-dependent glyceraldehyde-3-phosphate dehydrogenase (GapN) can be used to replace the native NAD-dependent isoenzyme for NADPH regeneration with improved carbon conservation^52–54^. However, GapN catalyzes the conversion of glyceraldehyde-3-phosphate (G3P) to glycerate-3-phosphate with an NADPH formation but no ATP formation. Therefore, the GapN-based pathway produces less ATP than the native GapA-Pgk (NAD-dependent glyceraldehyde-3-phosphate dehydrogenase and phosphoglycerate kinase) pathway that produces an NADH and an ATP (Fig. 2). By combining the GapN-based G3P oxidation pathway and the previously described Pyc-based C4 anaplerotic pathway, it is possible to provide sufficient ATP and NADPH for cell growth and l-proline biosynthesis. After adding the GapN-catalyzed reaction to the model, a simulation suggests the l-proline yield from glucose increases from 0.86 mol/mol to 0.98 mol/mol (0.63 g/g) (Fig. 2 and Supplementary Data 5). The metabolic flux through the oxidative PPP decreases by 84.9%. The G3P flux is divided into the GapN and GapA-Pgk pathways by a ratio of 86.2% to 13.8%. Based on these analyses, l-proline biosynthetic genes (*gdh*, *proB*, *proA*, and *proC*), *pyc*, and *gapN* are flux-control genes and need to be finely tuned for enhanced l-proline production.

### Chromosomally transcriptional tuning of flux-control genes using tailored promoter libraries

**Fig. 4:**
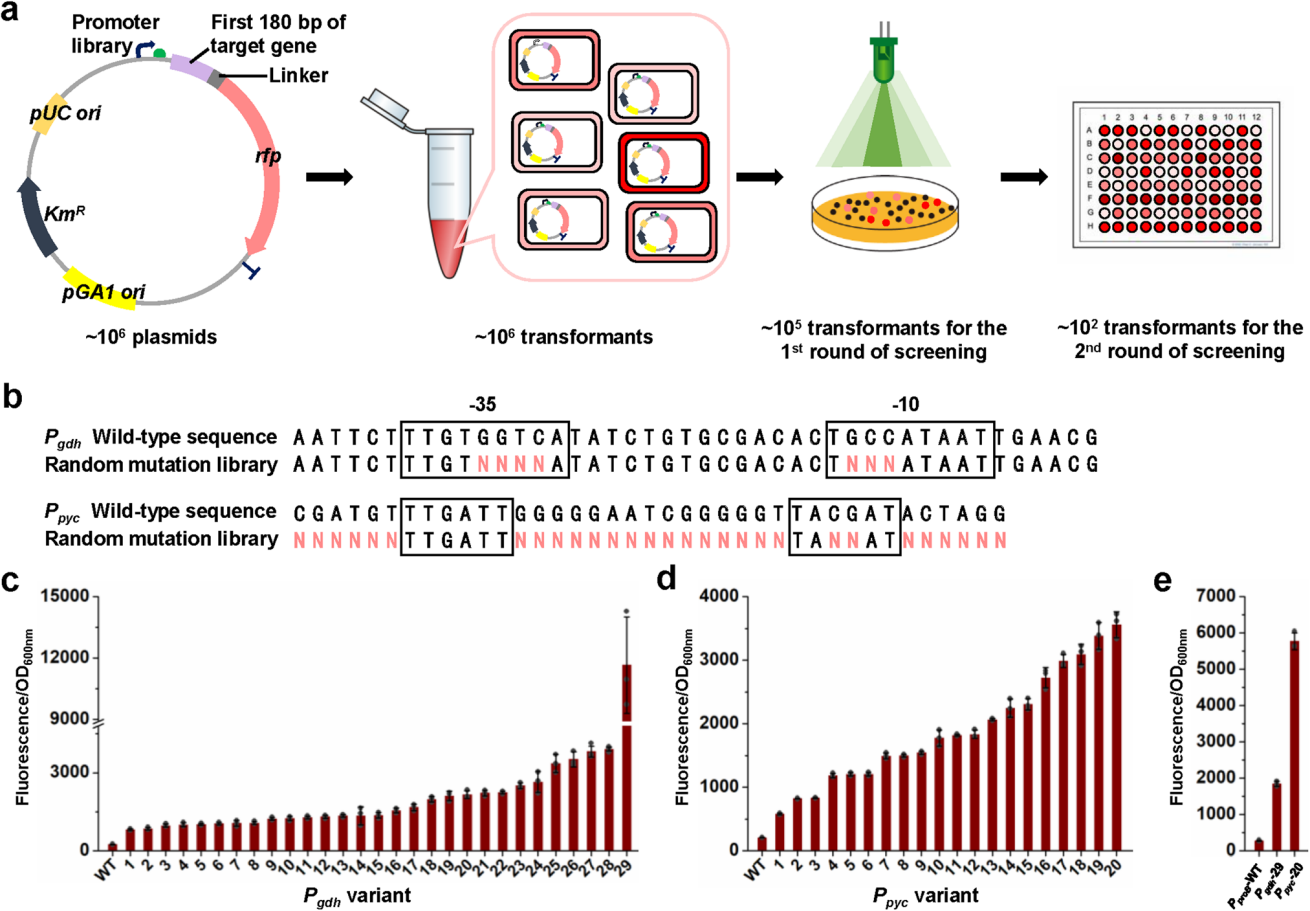
Construction and screening of tailored promoter libraries for target genes. **a** Workflow of construction and screening of tailored promoter libraries. The reporter gene was constructed by fusing the first 180 bp of the target gene (*gdh*, *pyc*, or *proB*) with a flexible linker (GGGGS)_3_ and an *rfp* gene. Random mutation libraries of native promoters were constructed by PCR with primers containing several degenerate bases. The plasmid libraries (~10^6^) were transformed into *C. glutamicum*. Approximately 1/10 transformants were first screened by colony fluorescence imaging and those with increased RFP fluorescence were cultivated in 96-deep-well plates for a second round of screening. **b** Representative *gdh* and *pyc* promoter libraries tested in this study. The sequences of wild-type promoter and random mutation library are shown. The degenerate bases introduced during PCR primer synthesis are highlighted in red. The predicted -35 and -10 regions are highlighted in black boxes. Other tested promoter libraries for *gdh*, *pyc*, and *proB* are shown in Supplementary Fig. 7. **c** Strength analysis of the *P*_*gdh*_ variants using the *gdh*-*rfp* fusion gene as a reporter. WT represents the wild-type *P*_*gdh*_ control. The number represents the serial number of *P*_*gdh*_ variants. Data are presented as mean values +/− SD (*n* = 3 independent experiments). **d** Strength analysis of the *P*_*pyc*_ variants using the *pyc*-*rfp* fusion gene as a reporter. WT represents the wild-type *P*_*pyc*_ control. The number represents the serial number of *P*_*pyc*_ variants. Data are presented as mean values +/− SD (*n* = 3 independent experiments). **e** Strength analysis of *P*_*gdh*_-29 and *P*_*pyc*_-20 using the *proB*-*rfp* fusion gene as a reporter. All the *P*_*proB*_ libraries failed to generate good diversity and the strongest *P*_*gdh*_-29 and *P*_*pyc*_-20 variants were used for expression of the *proB*-*rfp* fusion gene. The wild-type *P*_*proB*_ was used as a control. Data are presented as mean values +/− SD (*n* = 3 independent experiments). Cells of the stationary growth phase were used to detect their fluorescence outputs using a microplate reader (λ excitation = 560 nm, λ emission = 607 nm). Source data underlying Fig. 4c–e are provided as a Source Data file.

Promoter engineering is widely used to enhance gene expression at the transcription level. A number of homologous and heterologous promoters have been developed for constitutive or inducible gene expression in *C. glutamicum*^55^. However, the choice of strong and constitutive natural promoters is still limited^17^, and *P*_*sod*_ and *P*_*tuf*_ promoters are the mostly used ones for strain development^54,56^. Moreover, because the 5′ region of coding sequence (CDS) significantly effects gene expression^57,58^, a promoter usually shows different strengths for a reporter gene (e.g., a fluorescent protein-encoding gene) and the target gene, making it difficult to predict the promoter performance for metabolic tuning. To overcome this problem, we fused the first 180 bp of target gene with a flexible linker (GGGGS)_3_ and a red fluorescent protein (RFP) gene to construct a tailored reporter system. The native promoter of the target gene was used as a template to build promoter libraries for good adaptability to its naturally controlled gene (Fig. 4a). Regarding the screening method, fluorescence-activated cell sorting (FACS) is a commonly used high-throughput method for the first round of screening^59^. However, exponential reproduction of microbial cells during the cultivation of transformants may lower the proportion of variants that are overburdened by high-level protein expression^60^. To maximize the efficiency of the first round of screening, we used a fluorescence imaging system to directly assay the RFP fluorescence of 1/10 transformants on agar plates (~10^5^ colonies) (Fig. 4a and Supplementary Fig. 6). Cultivation in deep-well plates was used for the second round of screening of ~10^2^ colonies with enhanced RFP fluorescence.

Several libraries for native promoters of *gdh*, *proB*, and *pyc* were constructed by installing degenerate bases via chemical synthesis (Fig. 4b and Supplementary Fig. 7). The *P*_*gdh*_ library produced variants with 2.3- to 46.4-fold increases in the expression level of the tailored fluorescent reporter compared with the wild-type promoter. The *P*_*pyc*_ library covered 1.8- to 16.1-fold fluorescence increases compared with the original *P*_*pyc*_ (Fig. 4c and Supplementary Data 6). However, the construction of *P*_*proB*_ libraries failed to generate variants with stronger transcription initiation capabilities. Therefore, we tested the strongest promoter variants from the *P*_*gdh*_ and *P*_*pyc*_ libraries for the expression of the *proB* reporter. *P*_*gdh*_-29 and *P*_*pyc*_-20 led to 6.7- and 20.8-fold increases in the expression level of the *proB* reporter, respectively. Although the strengths of *P*_*gdh*_-29 and *P*_*pyc*_-20 for the *proB* reporter were different from those for their corresponding reporters, these two promoters can be used to enhance the expression of *proB* (Fig. 4c).

**Fig. 5:**
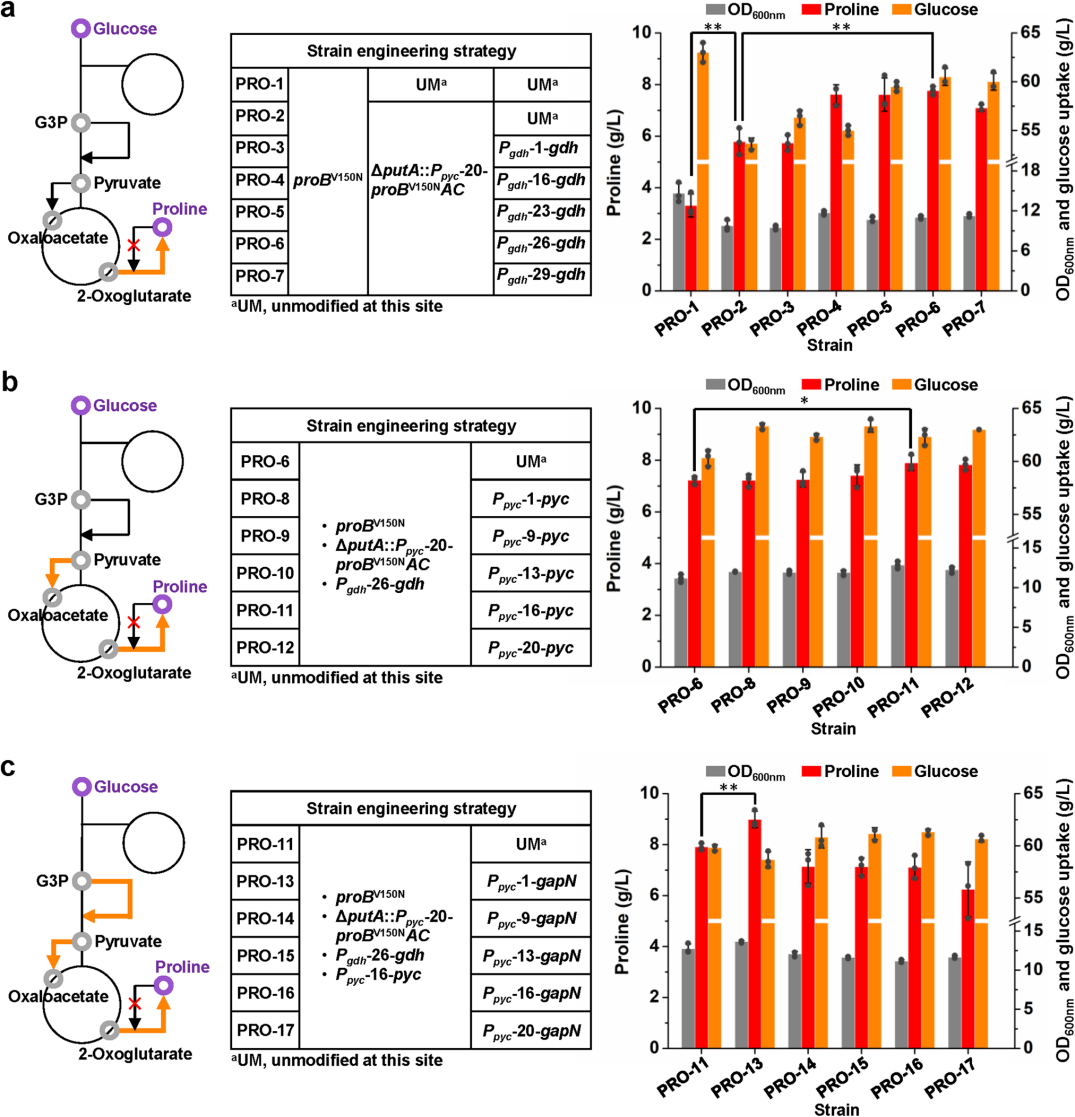
Enhancing l-proline production by tuning the expression of flux-control genes. **a** Channeling metabolic flux into l-proline biosynthesis by overexpressing an artificial *proB*^V150N^*AC* operon and *gdh*. Strain PRO-1 was constructed by introducing a V150N mutation to the *proB* gene in the chromosome of wild-type *C. glutamicum* ATCC 13032. Strain PRO-2 was constructed by inserting an artificial *proB*^V150N^*AC* operon in to the *putA* gene of strain PRO-1. Strains PRO-3 to PRO-7 were constructed by replacing the wild-type *P*_*gdh*_ promoter with different *P*_*gdh*_ promoter variants for *gdh* overexpression in strain PRO-2. l-Proline production was conducted in 24-deep-well plates. Data are presented as mean values +/− SD (*n* = 3 independent experiments). **P = 0.0032 for PRO-1 vs. PRO-2, **P = 0.0031 for PRO-2 vs. PRO-6, Student’s two-tailed *t*-test. **b** Strengthening C4 anaplerotic pathway by overexpressing *pyc*. Strains PRO-8 to PRO-12 were constructed by replacing the wild-type *P*_*pyc*_ promoter with different *P*_*pyc*_ promoter variants for *pyc* overexpression in strain PRO-6. l-Proline production was conducted in 24-deep-well plates. Data are presented as mean values +/− SD (*n* = 3 independent experiments). *P = 0.0260, Student’s two-tailed *t*-test. **c** Switching glycolysis cofactor from NADH to NADPH by overexpressing *gapN* from *S. mutans*. Strains PRO-13 to PRO-17 were constructed by introducing *gapN* downstream the *cgl2334* gene under the control of different *P*_*pyc*_ promoter variants in strain PRO-11. l-Proline production was conducted in 24-deep-well plates. Data are presented as mean values +/− SD (*n* = 3 independent experiments). **P = 0.0059, Student’s two-tailed *t*-test. Source data underlying Fig. 5 are provided as a Source Data file.

Next, the flux-control reactions were tuned using the screened promoter variants. All the genetic modifications were conducted in the *C. glutamicum* chromosome using the optimized CRISPR/Cas9-assisted genome editing method. First, based on strain PRO-1 expressing a chromosomal copy of *proB*^V150N^, an artificial *proB*^V150N^*AC* operon controlled by *P*_*pyc*-20_ was inserted into the *putA* locus for simultaneous enhancement of l-proline biosynthesis and disruption of l-proline degradation, resulting in strain PRO-2 (Supplementary Fig. 8). *putA* encodes the multifunctional l-proline utilization A flavoenzyme that catalyzes the oxidation of l-proline to l-glutamate in two reaction steps^61^ (Fig. 2). Deletion of *putA* has shown a beneficial effect for l-proline production^24^. Insertion of *proB*^V150N^*AC* operon into the *putA* locus resulted in a 75.8% increase in l-proline production (from 3.3 g/L to 5.8 g/L) in 24-deep-well plate cultivation (Fig. 5a). Meanwhile, biomass production and glucose consumption largely decreased, leading to a 120% increase in the conversion yield (from 0.05 g/g to 0.11 g/g). Second, *gdh* responsible for competition with TCA cycle for 2-oxoglutarate flux was overexpressed in strain PRO-2 by in-situ replacing the original *P*_*gdh*_ by its variants with gradually increased strengths (*P*_*gdh*_-1, *P*_*gdh*_-16, *P*_*gdh*_-23, *P*_*gdh*_-26, and *P*_*gdh*_-29, with 2.3-, 5.3-, 9.2-, 13.3-, and 46.4-fold strength increases, respectively). Strain PRO-6 in which *gdh* was overexpressed using *P*_*gdh*_-26 showed the largest improvement in l-proline production compared with strain PRO-2 (34.5% and 18.5% improvement in titer and yield, respectively) (Fig. 5a). The use of the strongest promoter *P*_*gdh*_-29 slightly decreased l-proline titer, which was possibly due to the trade-offs between the resources used for maintaining cellular metabolism and synthesizing l-proline. Third, the C4 anaplerotic pathway was enhanced by in-situ overexpressing *pyc* using *P*_*pyc*_ variants with gradually increased strengths (*P*_*pyc*_-1, *P*_*pyc*_-9, *P*_*pyc*_-13, *P*_*pyc*_-16, and *P*_*pyc*_-20, with 1.8-, 6.4-, 8.9-, 12.1-, and 16.1-fold strength increases, respectively). Moderate improvement in l-proline production was observed. Strain PRO-11 overexpressing *pyc* with the *P*_*pyc*_-16 variant produced 9.7% more l-proline with 5.8% higher yield compared with its parent strain PRO-6 (Fig. 5b). Finally, the NADPH-generating glycolysis branch was introduced to strain PRO-11 by expressing *gapN* from *Streptococcus mutans* using five *P*_*pyc*_ variants (*P*_*pyc*_-1, *P*_*pyc*_-9, *P*_*pyc*_-13, *P*_*pyc*_-16, and *P*_*pyc*_-20). By using a tailored reporter consisting of the first 180 bp of *gapN* and an RFP gene, it was observed that the five *P*_*pyc*_ variants led to gradual expression levels of *gapN* (Supplementary Fig. 9). The strength of *P*_*pyc*_ variants for expressing *gapN* was different from that for expressing its native *pyc* gene, which was reasonable considering the effects of the 5′ region of CDS as discussed previously^57,58^. Only expression of *gapN* with *P*_*pyc*_-1 (strain PRO-13) improved l-proline production. The titer and yield of strain PRO-13 reached 9.0 g/L and 0.15 g/g, which were 13.9% and 15.9% higher than its parent strain PRO-11, respectively. Use of stronger *P*_*pyc*_ variants caused growth retardation, suggesting very high-level expression of *gapN* negatively affected cellular metabolism (Fig. 5c).

### Identification of an l-proline exporter by arrayed CRISPRi screening

**Fig. 6:**
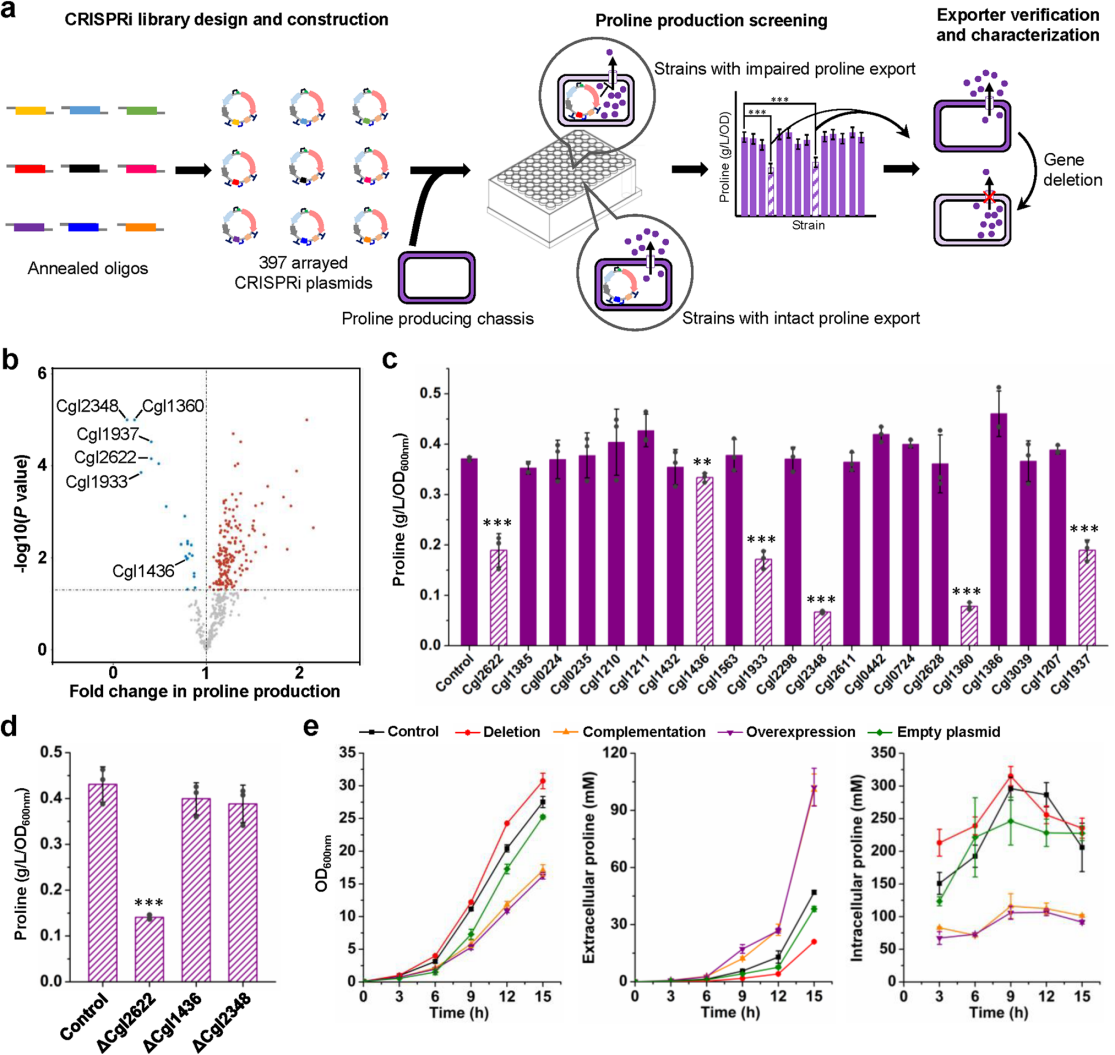
CRISPRi screening and characterization of l-proline exporter in *C. glutamicum*. **a** Workflow of screening l-proline exporter by constructing an arrayed CRISPRi library targeting the potential transporter genes. A pair of oligos were synthesized and annealed to generate dsDNAs harboring a spacer sequence. Totally 397 CRISPRi plasmids were constructed using the dsDNAs and pdCas9gRNA-*ccdB*. Each CRISPRi plasmid targeted a potential transporter gene of *C. glutamicum*. The CRISPRi plasmids were individually transformed into an l-proline producing *C. glutamicum* PRO-CRISPRi expressing a deregulated ProB^G149D^ variant^24^. The resultant 392 strains were cultivated in 96-deep-well plates for the first round of screening. Strains with significantly reduced l-proline production were selected for a second round of screening by cultivation in 24-deep-well plates. The transporter genes whose repression significantly reduced l-proline production were considered as potential l-proline exporters for characterization. **b** Volcano plot of differential l-proline production levels caused by CRISPRi repression of transporter genes. The results of the first round of screening are analyzed and shown. Three parallel l-proline production experiments were conducted in 96-deep-well plates for all the 392 strains. Strain PRO-CRISPRi harboring a nontargeting CRISPRi system was used as a control. Gene repressions causing significant increases and decreases in l-proline production are indicate in red and blue dots, respectively (*P* < 0.05, Student’s two-tailed *t*-test). Grey dots represent those with non-significant changes in l-proline production level. Twenty-one exporters (blue dots) were selected for a second round of screening. **c** The second round of screening of the 21 l-proline exporter candidates by cultivation in 24-deep-well plates. Data are presented as mean values +/− SD (*n* = 3 independent experiments). All Student’s two-tailed *t*-tests compare the l-proline production levels of strains expressing a gene-targeting CRISPRi system with the control strain expressing a nontargeting CRISPRi system (***P = 0.0007 for Cgl2622, **P = 0.0027 for Cgl1436, ***P = 0.00005 for Cgl1933, ***P = 3 × 10^−8^ for Cgl2348, ***P = 4 × 10^−7^ for Cgl1360, ***P = 0.0001 for Cgl1937). **d** Effects of deletion of *cgl2622*, *cgl1436*, and *cgl2348* on l-proline production. Strains were cultivated in 24-deep-well plates for l-proline production. Data are presented as mean values +/− SD (*n* = 3 independent experiments). ***P = 0.00003, Student’s two-tailed *t*-test. **e** Effects of deletion, complementation, and overexpression of *cgl2622* on growth and extracellular and intracellular accumulation of l-proline. Complementation was conducted by expressing *cgl2622* via a plasmid under the control of IPTG-inducible *P*_*trc*_ in the *cgl2622*-deleted PRO-CRISPRi strain. Strains were cultivated in shake flasks to obtain enough cells for the measurement of intracellular l-proline. Data are presented as mean values +/− SD (*n* = 3 independent experiments). Source data underlying Fig. 6b–e are provided as a Source Data file.

Chromosomally transcriptional tuning of flux-control genes improved l-proline production by nearly 3-fold. Besides metabolic pathways, transport systems are also pivotal for the hyperproduction of biochemicals^26^. To test whether l-proline excretion is a limiting step for its production, the intracellular l-proline concentration of strain PRO-13 during batch fermentation in shake flasks was measured and found to be 300–400 mM (Supplementary Fig. 10), which is almost 10-fold higher than the wild-type nonproducing strain^62^. Therefore, strengthening l-proline excretion is required for further strain improvement. As a compatible solute essential for protection against hyperosmotic shock, uptake systems of l-proline have been well characterized in microorganisms^63^, whereas l-proline exporter that transports the intracellularly synthesized l-proline molecules to the culture medium has not been discovered yet^25^. By using TransportDB 2.0^64^, *C. glutamicum* was predicted to possess nearly 400 membrane transporters (Supplementary Data 7). For a comprehensive analysis of *C. glutamicum* transporters and identification of l-proline exporters, we adopted CRISPRi technology for *C. glutamicum* and constructed an arrayed CRISPRi library consisting of 397 plasmids, each of which was designed to repress the transcription of a membrane transporter gene. The plasmid library was then transformed into an l-proline producing *C. glutamicum* chassis PRO-CRISPRi (*C. glutamicum* expressing a deregulated ProB^G149D^ variant^24^) to evaluate the effect of gene repression on l-proline biosynthesis. Transformation of five plasmids failed to produce any transformants possibly because repression of these genes seriously inhibited cell growth. Because impaired amino acid excretion could significantly decrease amino acid production^30^, the transporters whose repression brought significant decrease in l-proline production were considered as potential l-proline exporters and further characterized (Fig. 6a).

Two rounds of screening were conducted for identifying potential l-proline exporters. The first round of screening was performed by cultivating all the 392 strains with transporter-targeting CRISPRi systems and a negative control with a nontargeting CRISPRi system. Repression of twenty-one transporters were found to negatively affect l-proline production (Fig. 6b), which were subjected to a second round of screening by cultivation in 24-deep-well plates. After two rounds of screening, repression of six transporters (Cgl2622, Cgl1436, Cgl1933, Cgl2348, Cgl1360, and Cgl1937) decreased l-proline production (Fig. 6c). Cgl1933 (PtsI), Cgl1360 (PtsG), and Cgl1937 (PtsH) belong to the PTS for sugar uptake^65^, repression of which would hinder glucose uptake and consequently decrease l-proline production. Therefore, these three genes were not further investigated here. *cgl2622*, *cgl1436*, and *cgl2348* were individually deleted in strain PRO-CRISPRi by CRISPR/Cas9 to verify their functions. While CRISPRi of *cgl1436* slightly decreased l-proline production, its gene deletion showed no significant affect. Interestingly, the strain with repression of *cgl2348* only produced ~20% l-proline compared with the control without CRISPRi. However, deletion of *cgl2348* did not decrease l-proline production significantly. Cgl2348 was annotated as a DNA uptake protein or related DNA-binding protein. According to a previous transcriptomic analysis, *cgl2348* belongs to an operon containing *proB* and *proA* involved in l-proline biosynthesis^66^. CRISPRi of *cgl2348* may also affect the expression of its neighboring genes including *proB* and *proA*, leading to decreased l-proline production. Partial deletion of the CDS of *cgl2348* should not affect the expression of its neighboring genes, which could explain why CRISPRi and deletion of *cgl2348* showed different influences on l-proline production. Only deletion of *cgl2622* significantly decreased l-proline production, making Cgl2622 a candidate for l-proline exporter (Fig. 6d).

To verify the function of Cgl2622 as an l-proline exporter, two experiments were conducted. First, *cgl2622* was deleted, complemented, and overexpressed in the l-proline producing strain PRO-CRISPRi to test the effects on intracellular and extracellular accumulation of l-proline. The resultant strains were cultivated in shake flasks to obtain enough cells for the measurement. In strain PRO-CRISPRi, the intracellular l-proline accumulated to very high levels of 150–300 mM (Fig. 6e). Deletion of *cgl2622* further increased the intracellular l-proline concentration especially in the early phase of fermentation and largely decreased extracellular accumulation of l-proline. By complementing or overexpressing *cgl2622* in a plasmid with IPTG-inducible promoter *P*_*trc*_, intracellular l-proline concentration was maintained at relatively low levels between 50 mM to 100 mM, while extracellular l-proline production was largely increased (Fig. 6e). The similar results for complementation and overexpression of *cgl2622* are possibly because of the high-level expression of *cgl2622* using multi-copy plasmid and strong IPTG-inducible promoter. The results suggest that Cgl2622 plays an important role in maintaining the intracellular l-proline homeostasis and transporting l-proline outside cells.

Second, peptide uptake and amino acid export assay was conducted. *cgl2622* was deleted and complemented in *C. glutamicum* wild-type strain. Upon the addition of Thr-Pro peptide, the *cgl2622*-deleted mutant showed higher intracellular l-proline level and dramatically decreased l-proline export rate (Supplementary Fig. 11). Conversely, complementation of *cgl2622* in a plasmid with IPTG-inducible promoter *P*_*trc*_ largely decreased intracellular l-proline concentration but accelerated l-proline export (Supplementary Fig. 11). These results further verify the function of Cgl2622 as an l-proline exporter.

### Transport engineering to enhance l-proline production

**Fig. 7:**
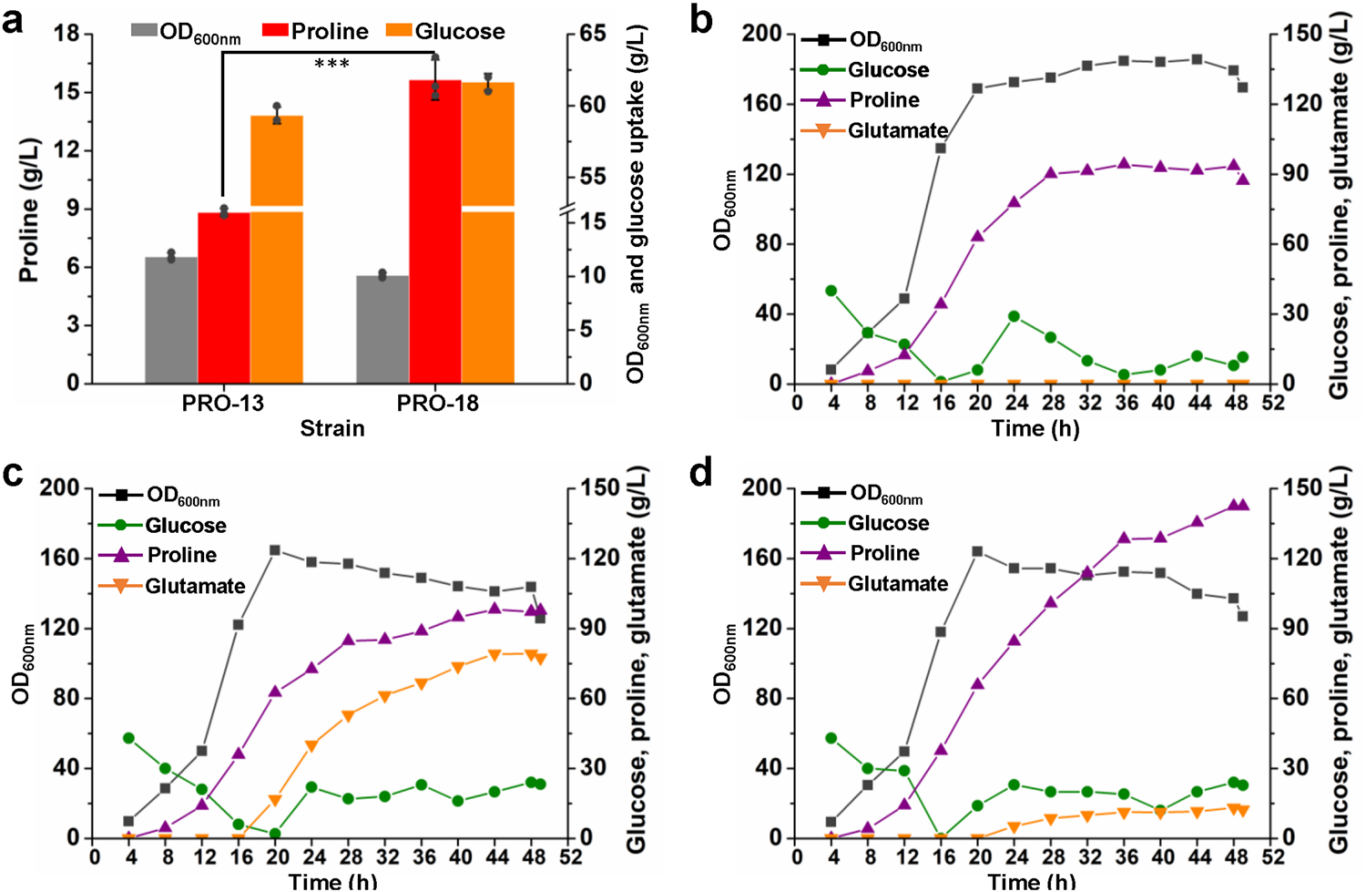
l-Proline production using strains with transport engineering. **a** Effects of *cgl2622* overexpression on l-proline production in 24-deep-well plates. Strain PRO-18 was constructed by adding a second copy of *cgl2622* controlled by the derepressed *P*_*trc*_ promoter in the chromosome of strain PRO-13. Data are presented as mean values +/− SD (*n* = 3 independent experiments). ***P = 0.0004, Student’s two-tailed *t*-test. **b** Fed-batch fermentation of strain PRO-18 in a 5 L bioreactor with 1 mg/mL biotin for a biotin-rich condition. **c** Fed-batch fermentation of strain PRO-18 in a 5 L bioreactor with 45 μg/mL biotin for a biotin-limited condition. **d** Fed-batch fermentation of strain PRO-19 (strain PRO-18 with *cgl1270* (*mscCG*) deleted) in a 5 L bioreactor. Biotin was used at 45 μg/mL for a biotin-limited condition. Source data underlying Fig. 7 are provided as a Source Data file.

To overcome the limitation of l-proline excretion in engineered producing strains, overexpression of Cgl2622 is required. To quantify the expression level of Cgl2622, we used the developed strategy of building a tailored fluorescent reporter by fusing the first 180 bp of *cgl2622* with the flexible linker (GGGGS)_3_ and fluorescent protein-encoding gene. Both RFP and GFP were tested, whereas the fusion protein failed to produce fluorescence, which was possibly due to the membrane topology of Cgl2622. Therefore, we directly added a second copy of *cgl2622* controlled by the derepressed *P*_*trc*_ promoter in the chromosome of strain PRO-13 for overexpression. The resultant strain PRO-18 produced 15.7 g/L l-proline with 0.25 g/g yield, which are 177.4% and 170.5% of the production level of its parent strain PRO-13 (Fig. 7a). Overexpression of Cgl2622 also led to ~100% decrease in intracellular l-proline concentration (Supplementary Fig. 12), which was considered as a major cause of improved l-proline production.

Fed-batch fermentation in a 5 L bioreactor was then conducted to evaluate the l-proline production by strain PRO-18. After 49 h cultivation, 87.2 g/L l-proline was produced with a productivity of 1.78 g/L/h and a yield of 0.19 g/g (Fig. 7b). The biomass reached a OD_600nm_ value over 180. It seemed that too much carbon flux was directed to the production of biomass rather than the target molecule l-proline. Since *C. glutamicum* is a biotin-auxotrophic bacterium^67^, biotin limitation can be used to balance the biosynthesis of biomass and target molecules. Therefore, a biotin-limited fed-batch fermentation was conducted. Although biomass formation was reduced, l-proline production was only moderately improved to 97.8 g/L with a productivity of 2.00 g/L/h and a yield of 0.23 g/g. Product analysis showed the high-level accumulation of extracellular l-glutamate up to 77.5 g/L (Fig. 7c).

### Improving l-proline production by eliminating biotin limitation-induced l-glutamate excretion

l-Glutamate production by *C. glutamicum* can be induced by treatments such as biotin limitation, Tween 40 addition, and penicillin addition. Biotin limitation decreases the activity of 2-oxoglutarate dehydrogenase complex (ODHC) that converts 2-oxoglutarate to succinyl-CoA, causing accumulation of intracellular 2-oxoglutarate. Meanwhile, the cell membrane tension is altered and mechanosensitive ion channels responsible for l-glutamate excretion are activated^30,67^. To reduce l-glutamate production, the major l-glutamate exporter MscCG encoded by *cgl1270* was deleted in strain PRO-18 by CRISPR/Cas9. The resultant strain PRO-19 was then used for l-proline production in biotin-limited fed-batch fermentation. After 49 h cultivation, 142.4 g/L l-proline was produced with a productivity of 2.90 g/L/h and a yield of 0.31 g/g (Fig. 7d). The titer, productivity, and yield of strain PRO-19 were improved by 44.9, 45.0, and 34.8%, respectively, compared with strain PRO-18 under the same cultivation condition. The byproduct l-glutamate was largely decreased to 12.3 g/L, which was only 15.6% of that produced by strain PRO-18. The amino acid profile of the broth from the end of fermentation was further determined using an amino acid analyzer. Besides l-glutamate, 7.9 g/L of l-alanine and 3.2 g/L of l-valine were also detected as byproducts (Supplementary Fig. 13). Because the l-glutamate exporter MscCG encoding gene *cgl1270* was deleted in strain PRO-19, l-glutamate may accumulate inside the cells. l-Glutamate is an important amine donor for the biosynthesis of many amino acids^68^. Its accumulation may enhance the transamination and biosynthesis of other amino acids, which may explain the formation of l-alanine and l-valine as byproducts. Therefore, increasing the expression of genes involved in l-proline biosynthesis (such as *gdh* and *proB*) and deleting related aminotransferase encoding genes may further improve l-proline production and decrease byproduct formation.

## Discussion

To develop industrial strains for biomanufacturing, efficient genetic manipulation tools and multifarious components with catalytic, regulatory, or transport functions are needed. Here, CRISPR/Cas9-assisted rational flux-tuning and CRISPRi-mediated comprehensive transporter discovery were applied to transfer the wild-type *C. glutamicum* strain to an industrial-strength l-proline producer. Taking advantage of the discovered l-proline exporter Cgl2622, the titer, yield, and productivity of the final strain PRO-19 are superior to those of most speciality amino acids (Supplementary Table 1). No plasmid, antibiotic marker, or chemically inducible promoter is contained in the final strain, ensuring the stable production performance in scale-up fermentations.

Both the CRISPR/Cas9^31–34^ and CRISPR/Cas12a^35–38^ systems have been adopted for genetic manipulation of *C. glutamicum*. Considering the capability of accepting G-rich PAMs and distinguishing a single nucleotide mismatch^40^, the CRISPR/Cas9 system was selected and optimized for genome editing of *C. glutamicum* in this study. By combating the cytotoxicity of CRISPR/Cas9 through strict control of *cas9* expression, the optimized method showed satisfactory performance in codon saturation mutagenesis, biosynthetic operon integration, and in-situ promoter replacement, which lays the technical groundwork for the development of high-performance strains for the production of l-proline and other biobased chemicals. Previous studies suggest that the CRISPR/Cas12a system is less toxic and facilitates efficient genome editing in *C. glutamicum*^35–38^. Cas12a possesses both DNase and RNase activities and thus it can process a CRISPR RNA array into multiple mature gRNAs, providing unique benefits for multiplex editing^69^. Since the CRISPR/Cas12a system recognizes T-rich PAMs that are different from the CRISPR/Cas9 system^39^, they can be complementary tools for the engineering of *C. glutamicum*.

Since microorganisms are commonly evolved for converting carbon sources to biomass, metabolic flux redirection is essential for hyperproduction of target chemicals^70^. We performed in silico FBA using a genome-scale metabolic model of *C. glutamicum*^24^ to predict the flux-control reactions in l-proline biosynthesis. The l-proline biosynthetic pathway, C4 anaplerotic pathway, and NADPH-generating glycolytic pathway were suggested as the key targets for engineering. Overexpression of deregulated *proB* and *gdh* is obviously important for l-proline overproduction^23^. Fixation of CO_2_ via the C4 anaplerotic pathway has been proven a useful strategy for improving the production yield of several chemicals, especially the l-aspartate family amino acids such as l-lysine^50,71^. Here, we demonstrate that the biosynthesis of l-proline also benefits from the enhanced C4 anaplerotic pathway. In previous studies, increasing the supply of NADPH for l-proline biosynthesis was commonly accomplished by strengthening the PPP^23^. To decrease CO_2_ release and increase l-proline yield, the in silico simulation suggests a PPP-independent alternative for NADPH regeneration based on the nonphosphorylating NADP-dependent G3P dehydrogenase (GapN). GapN was initially used for improving l-lysine biosynthesis that requires 4 mol NADPH/mol l-lysine. It was found that cell growth on glucose was significantly retarded by the complete replacement of the native NADH-generating glycolytic pathway with the GapN-based NADPH-generating glycolytic pathway^52^. Since the in silico simulation also suggests the collaboration between these two glycolytic pathways, GapN was expressed using different promoters in this study to achieve the balance between them. These efforts for flux-tuning led to a nearly 3-fold improvement in l-proline titer compared with the strain only harboring a feedback-insensitive γ-glutamyl kinase variant. Besides the catalytic reactions inside the cell, transport reactions that happen on the membrane also influence flux distribution by providing a strong driving force for continuous biosynthesis^72,73^. Because exporters for l-proline have not been discovered yet, transport engineering was not applied for l-proline production in previous studies. We constructed an arrayed CRISPRi library targeting all the 397 potential membrane transporter genes for a comprehensive screening in *C. glutamicum*, and identified Cgl2622 as an l-proline exporter. Its overexpression largely enhanced l-proline production. During the CRISPRi screening, it was observed that repression of several transporter genes increased l-proline production, which provided interesting targets for further strain improvement (Fig. 6b). This arrayed CRISPRi library can be readily used to screen undiscovered transporters for other metabolites of interest, such as the unavailable exporters for glycine, l-glutamine, and l-asparagine^25,26^. The results demonstrate that arrayed CRISPRi screening is a useful strategy to identify undiscovered functional components such as transporters.

Cgl2622 (previously known as ThrE) was previously characterized as an l-threonine exporter, which also accepted l-serine as a substrate but with a lower efflux rate^28^. Cgl2622 belongs to a ubiquitous ThrE family of putative transmembrane amino acid efflux that exists in both Gram-negative and Gram-positive bacteria, as well as in archaea and eukaryotes^74^. However, upon Cgl2622 overexpression and further metabolic engineering, very limited improvement in l-threonine production was observed^75^. Therefore, it is suggested that Cgl2622 is possibly not natively designed to export l-threonine but an ancient genomic relict using a still unknown compound as substrate^25^. Based on the observation that overexpression of Cgl2622 improved l-proline production by ~1.7-fold regarding both titer and yield, we hypothesize that l-proline is possibly the native export substrate for Cgl2622. It is no surprise that a transporter has more than one substrate. For instance, BrnFE of *C. glutamicum* exports branched-chain amino acids (l-leucine, l-isoleucine, and l-valine) and l-methionine^29,76^. Considering l-proline is one of the compatible solutes used by *C. glutamicum*^63^, Cgl2622 may cooperate with mechanosensitive ion channels for osmotic adaptation^30,77,78^. Studies on the expression regulation of Cgl2622 possibly provides clues to the biological functions of this ubiquitous transporter. Besides, the discovery of Cgl2622 as an l-proline exporter should be helpful for identifying l-proline exporters in other species.

In conclusion, we developed a highly efficient l-proline producing *C. glutamicum* strain using CRISPR-assisted metabolic flux-tuning and arrayed CRISPRi screening of the l-proline exporter Cgl2622. The optimized CRISPR/Cas9 system for genome editing, arrayed CRISPRi library for transporter genes, and interdisciplinary strategy for strain development will provide a pipeline for developing high-performance strains for biomanufacturing.

## Methods

### Strains and culture conditions

Strains used in this study are listed in Supplementary Data 8. *E. coli* strains Trans1-T1 and DB3.1 (TransGen Biotech, Beijing, China) used for plasmid cloning were cultivated aerobically at 37 °C in Luria–Bertani (LB) broth. Kanamycin (50 μg/mL) or chloramphenicol (20 μg/mL) was added to the medium as required. *C. glutamicum* ATCC 13032, ATCC 13869, and the derivatives were cultivated aerobically at 30 °C in TSB medium containing 5 g/L glucose, 9 g/L soya peptone, 5 g/L yeast extract, 1 g/L K_2_HPO_4_·3H_2_O, 0.1 g/L MgSO_4_·7H_2_O, 3 g/L urea, 0.5 g/L succinic acid, 10 μg/L biotin, 100 μg/L vitamin B1, and 20 g/L MOPS (pH 7.2). Kanamycin (25 μg/mL), chloramphenicol (5 μg/mL), or IPTG (0.01 to 0.5 mM) was added as required.

### Plasmid construction

Plasmids used in this study are listed in Supplementary Data 8. Plasmids were constructed via recombination or Golden Gate assembly. Recombination was conducted using the ClonExpress MultiS One Step Cloning Kit (Vazyme, Nanjing, China). Restriction endonucleases and T4 DNA ligase used for Golden Gate assembly were purchased from New England Biolabs (Beijing) (Beijing, China). DNA polymerase and reagents used for PCR were purchased from TransGen Biotech (Beijing, China). Services of primer and gene synthesis and DNA sequencing were provided by GENEWIZ Inc. (Suzhou, China). Primers and details for constructing plasmids are described in Supplementary Data 9.

### Genetic manipulation of *C. glutamicum*

For DNA deletion in *C. glutamicum*, 1 μg plasmid (for example pCas9gRNA-1 or pCas9gRNA-2) expressing Cas9, a targeting gRNA, and two HR arms was transformed into *C. glutamicum* via electroporation. Transformants were plated on TSB solid medium supplemented with 0.05 mM IPTG and 5 μg/mL chloramphenicol for counter-selection. After 24 h of cultivation at 30 °C, colonies were verified by PCR. For DNA insertion in *C. glutamicum*, 1 μg plasmid (for example pCas9gRNA-5) expressing Cas9 and a targeting gRNA and 1 μg plasmid (for example, pEC-1) harboring the inserted fragment and two HR arms were co-transformed into *C. glutamicum* via electroporation. Transformants were plated on TSB solid medium supplemented with 0.05 mM IPTG, 5 μg/mL chloramphenicol, and 25 μg/mL kanamycin for counter-selection. After 48 h of cultivation at 30 °C, colonies were verified by PCR. For ssDNA recombineering for codon saturation mutagenesis of CgProB, 1 μg plasmid pRecT expressing RecT was first transformed into *C. glutamicum* via electroporation. Transformants were plated on TSB solid medium supplemented with 25 μg/mL kanamycin. After 24 h of cultivation at 30 °C, colonies were verified by PCR. A correct transformant was used for preparation of competent cells and transformation of 1 μg plasmid (for example pCas9gRNA-6) expressing Cas9 and a targeting gRNA and 5 μg synthetic ssDNA (Supplementary Data 10). Transformants were plated on TSB solid medium supplemented with 0.05 mM IPTG, 5 μg/mL chloramphenicol, and 25 μg/mL kanamycin for counter-selection. After 18–24 h of cultivation at 30 °C, colonies were picked and cured for the plasmids. For plasmid curing, transformants were cultivated in TSB medium without antibiotics at 37 °C overnight and spread on TSB plates without antibiotics. Colonies were confirmed as cured by determining their sensitivity to antibiotics. Transformants with plasmid cured were used for the next round of genetic manipulation or other tests. An operation scheme of CRISPR/Cas9-assisted gene deletion, insertion, and ssDNA recombineering is shown in Supplementary Fig. 4. For gene overexpression in the plasmid, the target gene was amplified and inserted into the pEC-XK99E plasmid under the control of IPTG-inducible promoter *P*_*trc*_. The recombinant plasmid was transformed into *C. glutamicum* via electroporation. Gene overexpression in the transformant was induced by the addition of 0.05 mM IPTG. All the plasmids and gRNAs used for genetic manipulation of *C. glutamicum* are shown in Supplementary Data 8 and Supplementary Data 11, respectively.

Electro-competent cells for *C. glutamicum* wild-type strains and low-level l-proline producing strains PRO-1 and PRO-CRISPRi were prepared using a hypertonic medium containing glycine and dl-threonine for weakening cell walls and Tween 80 and isonicotinic acid hydrazide for increasing cytoplasmic membrane fluidity^79^. Electro-competent cells for high-level l-proline producing strain PRO-2 and its derivatives were prepared using a modified procedure because of the low growth rate caused by l-proline overproduction. Cells were cultivated overnight in TSB medium and transferred into 100 mL fresh TSB medium supplemented with 3% glycine and 0.1% Tween 80 to an OD_600nm_ value of 0.3 for aerobic cultivation at 30 °C. When the OD_600nm_ of culture reached ~0.8, cells were collected by centrifugation at 6,000 × *g* and 4 °C for 10 min After washed with ice-cold deionized distilled water for four times, cells were resuspended in 0.5 mL 10.0% (v/v) glycerol and 100 μL aliquots of competent cells were obtained. DNA (less than 10 μL) was added to the competence cells and transferred to a 2 mm electroporation cuvette (Bio-Rad Laboratories (Shanghai), China). Electroporation was performed with the Eppendorf Electroporator 2510 (Eppendorf China, Shanghai, China) with parameters set at 2,500 V and 5 ms. After electroporation, 1 mL TSB medium was added immediately and the suspension was quickly incubated for 6 min at 46 °C. Cells were incubated for 3 h at 30 °C, and spread on TSB plates supplemented with antibiotics and IPTG as required. The plates were incubated at 30 °C until colonies appeared.

### Homologous modeling of CgProB

The model structure of CgProB was constructed with the crystal structure of γ-glutamyl kinase from *E. coli* (PDB ID: 2J5T)^80^ as a template (93% coverage and 38% sequence identity with CgProB) using Discovery Studio 2018 software (Accelrys, USA). Molecular docking with l-glutamate and selection of the optimal conformation was performed using AutoDock Tools 1.5.6^81^. Receptor-ligand interaction analysis and figure rending were performed using Discovery Studio 2018 software (Accelrys, USA).

### In silico analysis of the optimal l-proline biosynthetic pathway

The genome-scale metabolic model *i*CW773^24^ was used to predict the optimal l-proline biosynthetic pathway in *C. glutamicum* by performing FBA^48^. Simulations were performed using the COBRApy toolbox (v 0.22.1)^82^. Uptake rate of glucose was set as 10 mmol/gCDW·h. To simulate the Ppc-based C4 anaplerotic pathway for l-proline biosynthesis, no modification to the model was needed. To simulate the Pyc-based C4 anaplerotic pathway for l-proline biosynthesis, the Ppc-catalyzed reaction was manually closed and oxaloacetate was synthesized from pyruvate via Pyc-catalyzed reaction. To simulate the GapN-Pyc-based pathway for l-proline biosynthesis, the GapN-catalyzed reaction was added to the model.

### Promoter library construction and screening

Native promoters of *gdh*, *proB*, and *pyc* were amplified from the genomic DNA of *C. glutamicum* ATCC 13032. The promoter was then ligated with the corresponding tailored RFP reporter gene and pEC-XK99E plasmid to construct a basic plasmid for library construction. The basic plasmid was used as a template for library construction via PCR with primers containing degenerate bases as shown in Fig. 4b and Supplementary Fig. 7. The PCR product was self-ligated and transformed into *E. coli* Trans1-T1. All the transformants were collected and used for plasmid extraction. The plasmid library was transformed into *C. glutamicum* via electroporation and cells were plated on TSB agar plates. After 24 h cultivation, the RFP fluorescences of 1/10 transformants on agar plates (~10^5^ colonies) were directly assayed using a Tanon 5200 Multi Fluorescence Imaging system (Tanon Technology, Shanghai, China). Deep-well plates were used for the second round of screening of ~10^2^ colonies with enhanced RFP fluorescence. Cells of the stationary growth phase were used to detect their fluorescence outputs using a microplate reader (SpectraMax M5, Molecular Devices, USA, λ excitation = 560 nm, λ emission = 607 nm).

### l-Proline production by cultivation in 24-deep-well plates

The seed medium (TSC medium) contains 5 g/L glucose, 3 g/L soya peptone, 1 g/L yeast extract, 1 g/L K_2_HPO_4_·3H_2_O, 0.1 g/L MgSO_4_·7H_2_O, 3 g/L urea, 0.5 g/L succinic acid, 10 μg/L biotin, 100 μg/L vitamin B1, and 20 g/L MOPS (pH 7.2). The fermentation medium (TSD medium) contains 80 g/L glucose, 1 g/L soya peptone, 1 g/L yeast extract, 1 g/L NaCl, 1 g/L (NH_4_)_2_SO_4_, 1 g/L K_2_HPO_4_·3H_2_O, 0.45 g/L MgSO_4_·7H_2_O, 0.05 g/L FeSO_4_·7H_2_O, 6 g/L urea, 400 μg/L biotin, 100 μg/L vitamin B1, and 40 g/L MOPS (pH 7.2). For seed preparation, strains were cultivated in 24-deep-well plates containing 800 μL TSC medium in each well at 30 °C, 800 rpm, and 90% moisture for 8 h. The seed culture was then used to inoculate 24-deep-well plates containing 800 μL TSD medium in each well to an initial OD_600nm_ of 0.06. The 24-deep-well plates were cultivated at 30 °C, 800 rpm, and 90% moisture for 18 h and cultivation was stopped for glucose and l-proline assay.

### l-Proline production by cultivation in shake flasks

TSC medium was used for seed preparation. The fermentation medium is TSD medium with urea changed from 6 g/L to 10 g/L for providing more nitrogen source for cell growth and l-proline production. For seed preparation, strains were cultivated in 250 mL shake flasks containing 25 mL TSC medium at 30 °C and 220 rpm for 8 h. The seed culture was then used to inoculate 250 mL shake flasks containing 25 mL TSD medium (with 10 g/L urea) to an initial OD_600nm_ of 0.1. The shake flasks were cultivated at 30 °C and 220 rpm for 15 h and cultivation was stopped for glucose and l-proline assay.

### l-Proline production by cultivation in 5 L bioreactors

TSB medium was used for seed preparation. The fermentation medium contains 50 g/L glucose, 10 g/L corn steep powder, 10 g/L soya peptone, 10 g/L bovine bone peptone, 30 g/L (NH_4_)_2_SO_4_, 6 g/L KH_2_PO_4_, 0.5 g/L MgSO_4_·7H_2_O, 0.05 g/L FeSO_4_·7H_2_O, 0.03 g/L MnSO_4_·H_2_O, 45 or 1000 μg/L biotin, 45 μg/L vitamin B2, and 0.5 g/L antifoam. Fed-batch fermentations were conducted in 5 L bioreactors (Baoxing Bio., Shanghai, China). For seed preparation, strains were cultivated overnight in 500 mL shake flasks containing 75 mL TSB medium at 30 °C and 220 rpm. The seed culture was then used to inoculate 5 L bioreactors containing 1.5 L fermentation medium with an inoculation volume of 10%. Cultivation temperature was maintained at 30 °C, and pH was automatically controlled at 7.0 by the addition of NH_4_OH. The dissolved oxygen was maintained at 30% by automatic adjustment of agitation and aeration rate. Glucose solution (800 g/L) was fed into the bioreactor at appropriate rates to maintain the glucose concentration in the range of 5–30 g/L. Samples were withdrawn from the bioreactor every 4 h for measuring cell biomass, glucose, and products.

### CRISPRi library construction and screening

gRNAs targeting transporter genes of *C. glutamicum* were identified and analyzed for potential off-target sites using the sgRNAcas9 bioinformatics tool^83^. For each gRNA, a pair of 24-nt oligos were synthesized and annealed to generate dsDNAs harboring a spacer sequence. Golden Gate assembly strategy was applied to construct CRISPRi plasmid harboring gRNA targeting a specific gene using plasmid pdCas9gRNA-*ccdB* and the annealed dsDNA^84^. The predicted transporter genes and the corresponding gRNAs are shown in Supplementary Data 7. Totally 397 CRISPRi plasmids were constructed and individually transformed into an l-proline producing *C. glutamicum* with CgProB^G149K^ (strain PRO-CRISPRi). Plasmid and strain construction was conducted using the BioFoundry infrastructure at Tianjin Institute of Industrial Biotechnology and the developed automated Golden Gate assembly protocol^84^. The transformation of five plasmids failed to produce any colonies possibly because repression of these genes seriously inhibited cell growth. Two rounds of screening were conducted to identify the l-proline exporter. For the first round of screening, the resultant 392 strains with CRISPRi system targeting 392 potential transporter genes and a control strain with a nontargeting CRISPRi system were cultivated in 96-deep-well plates for l-proline production. Three replicates were conducted. For seed preparation, strains were cultivated in 96-deep-well plates containing 200 μL TSC medium in each well at 30 °C, 800 rpm, and 90% moisture for 8 h. Because determining and adjusting the OD_600nm_ values of seed cultures for all the tested strains is laborious, 3 μL of each seed culture was used to inoculate 96-deep-well plates containing 200 μL TSD medium in each well. Several wells were sampled to determine the initial OD_600nm_, which was approximately 0.06. The inoculated 96-deep-well plates were cultivated at 30 °C, 800 rpm, and 90% moisture for 21 h and cultivation was stopped for glucose and l-proline assay. IPTG (0.03 mM) was added to induce dCas9 expression. The strains that showed significant decrease in l-proline production were selected for a second round of screening in 24-deep-well plates. Three replicates were conducted. For seed preparation, strains were cultivated in 24-deep-well plates containing 800 μL TSC medium in each well at 30 °C, 800 rpm, and 90% moisture for 8 h. The OD_600nm_ values of seed cultures were detected and adjusted to the same value of 2.4 by dilution with TSC medium. To start the cultivation, 20 μL of the diluted seed culture was used to inoculate 24-deep-well plates containing 800 μL TSD medium in each well to an initial OD_600nm_ of 0.06. The 24-deep-well plates were cultivated at 30 °C, 800 rpm, and 90% moisture for 18 h and cultivation was stopped for glucose and l-proline assay. The target genes whose repression caused significant decrease in l-proline production were further analyzed.

### Peptide uptake and amino acid export assay

To determine the peptide uptake and amino acid export, *C. glutamicum* wild-type strain, *cgl2622*-deleted strain, and *cgl2622* complemented strain were cultivated overnight. Cells were used to inoculate CGXII medium^85^ containing 20 g/L glucose and 1 mM Thr-Pro peptide to an initial OD_600nm_ of 2.0. After incubation at 30 °C and 220 rpm for 2 h, cells were harvested by centrifugation at 5,000 × *g* for 10 min and resuspended in prewarmed CGXII medium (30 °C) containing 20 g/L glucose and 3 mM Thr-Pro peptide to an initial OD_600nm_ of 8.0. Cells were incubated at 30 °C and 220 rpm and samples were taken every 20 min Intracellular and extracellular l-proline concentrations were quantified using HPLC as described below.

### Analytical methods

Glucose in the medium was quantified using an SBA-40D biosensor analyzer (Institute of Biology of Shandong Province Academy of Sciences, Jinan, China). Extracellular l-proline concentrations of cultivation in deep-well plates and shake flasks were quantified according to the method based on the acid-ninhydrin reaction^86^. Extracellular amino acids of cultivation in 5 L bioreactors were quantified using a HPLC method and an L-8900 Amino Acid Analyzer (Hitachi, Japan). Cell cultures were centrifuged at 13,000 × *g* for 5 min, and the supernatant was used for detection after appropriate dilution. The HPLC system consists of a Prominence UFLC (Shimadzu, Japan) equipped with a Zorbax Eclipse AAA column (4.6 mm × 150 mm, 5 μm, Agilent Technologies, USA) and a UV detector^30^. A gradient of 50 mM sodium acetate buffer at pH 6.4 with a gradient solution containing acetonitrile-water (50%, v/v) was used as the eluent. Amino acids were detected as their 2,4-dinitrofluorobenzene derivatives at 360 nm by following the precolumn derivation method. The L-8900 Amino Acid Analyzer (Hitachi, Japan) was used to analyze the amino acid profile of the fermentation broth of strain PRO-19 using the amino acids mixture standard solutions (Type AN-2 and Type B, FUJIFILM Wako Pure Chemical Corporation, Japan) as standards. Analysis was performed according to the manufacturer’s instructions. Intracellular amino acids were extracted using a centrifugal separation procedure involving centrifugation of cells through a layer of silicon oil^87^ and then quantified using the abovementioned HPLC method. The intracellular volume used to calculate the internal amino acid concentration was 1.7 μL/mg DCW^88^.

### Statistics

Error bars indicate standard deviations from three parallel experiments. All *P* values were generated from two-tailed *t*-tests using the Microsoft Excel 2016 (Microsoft Corporation, USA).

### Reporting summary

Further information on research design is available in the Nature Research Reporting Summary linked to this article.

## Supplementary information

Supplementary Information

Description of Additional Supplementary Files

Supplementary Data 1

Supplementary Data 2

Supplementary Data 3

Supplementary Data 4

Supplementary Data 5

Supplementary Data 6

Supplementary Data 7

Supplementary Data 8

Supplementary Data 9

Supplementary Data 10

Supplementary Data 11

Reporting Summary

## Data Availability

The data supporting the findings of this work are available within the paper and the Supplementary Information files. A reporting summary for this article is available as a Supplementary Information file. The crystal structure of γ-glutamyl kinase from *E. coli* (PDB ID: 2J5T, www.rcsb.org/structure/2J5T) was used as a template for modeling of CgProB. Source data are provided with this paper.

## Source data

Source Data
