# Supplementary material for: CRISPR-assisted rational flux-tuning and arrayed CRISPRi screening of an l-proline exporter for l-proline hyperproduction
Source: Nat Commun. 2022 Feb 16;13:891. doi: 10.1038/s41467-022-28501-7 (PMC8850433; doi:10.1038/s41467-022-28501-7)
Supplement: Supplementary file 1 — Supplementary Information [file 41467_2022_28501_MOESM1_ESM.docx]

**Supplementary Information for**

**CRISPR-assisted rational flux-tuning and arrayed CRISPRi screening of an l-proline exporter for l-proline hyperproduction**

**Liu *et al.***


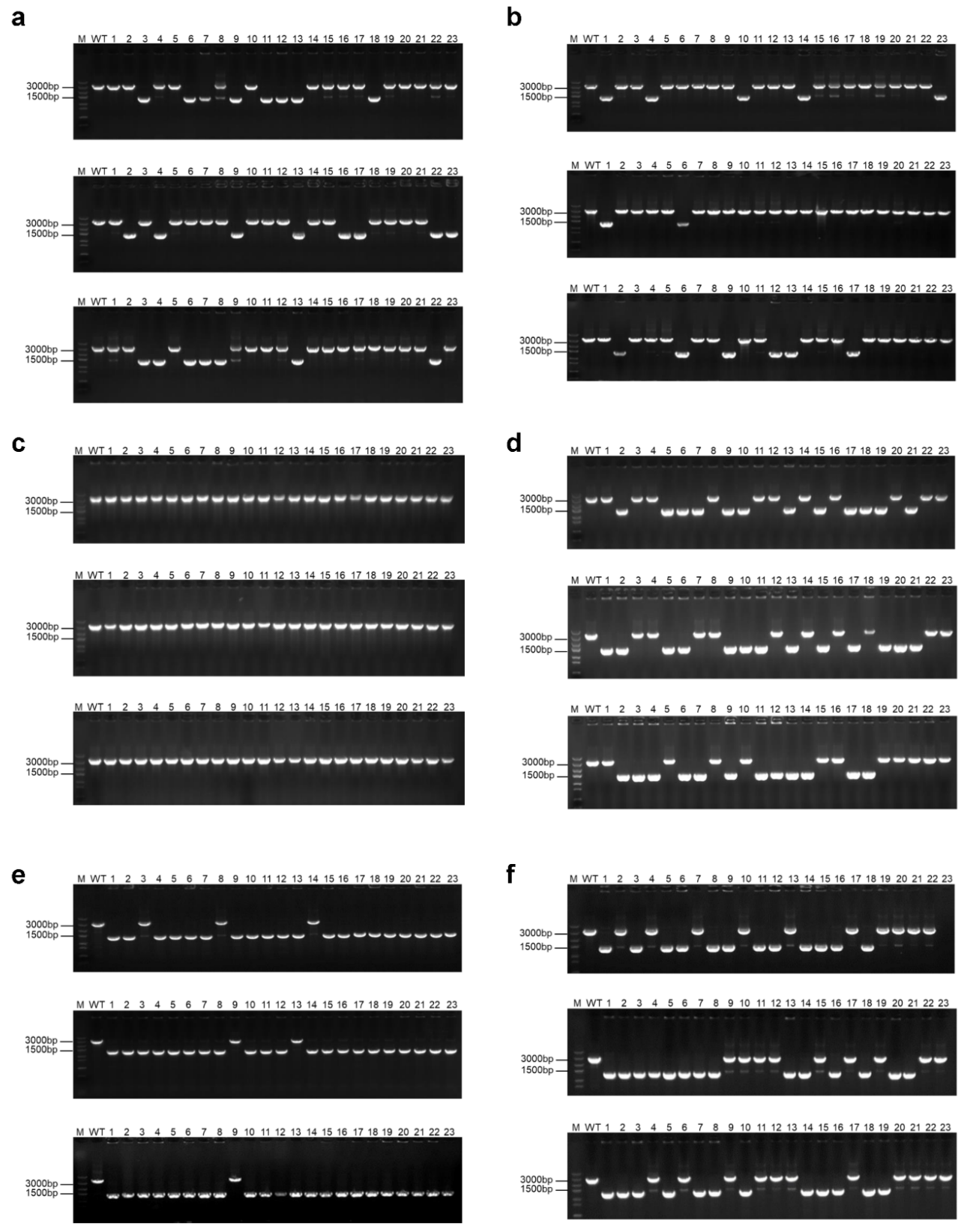


**Supplementary Fig. 1. PCR verification of CRISPR/Cas9-based gene deletion of a 1.7 kb DNA fragment in *C. glutamicum*.** **a**, Results for Fig. 1a, 0.01 mM IPTG. **b**, Results for Fig. 1a, 0.05 mM IPTG. **c**, Results for Fig. 1a, 0.5 mM IPTG. **d**, Results for Fig. 1b, 0.01 mM IPTG. **e**, Results for Fig. 1b, 0.05 mM IPTG. **f**, Results for Fig. 1b, 0.5 mM IPTG. The primers for colony PCR verification were designed at the upstream and downstream sites of the deleted fragment. Three independent replicates were conducted and twenty-three colonies were randomly selected and verified by PCR for each test.


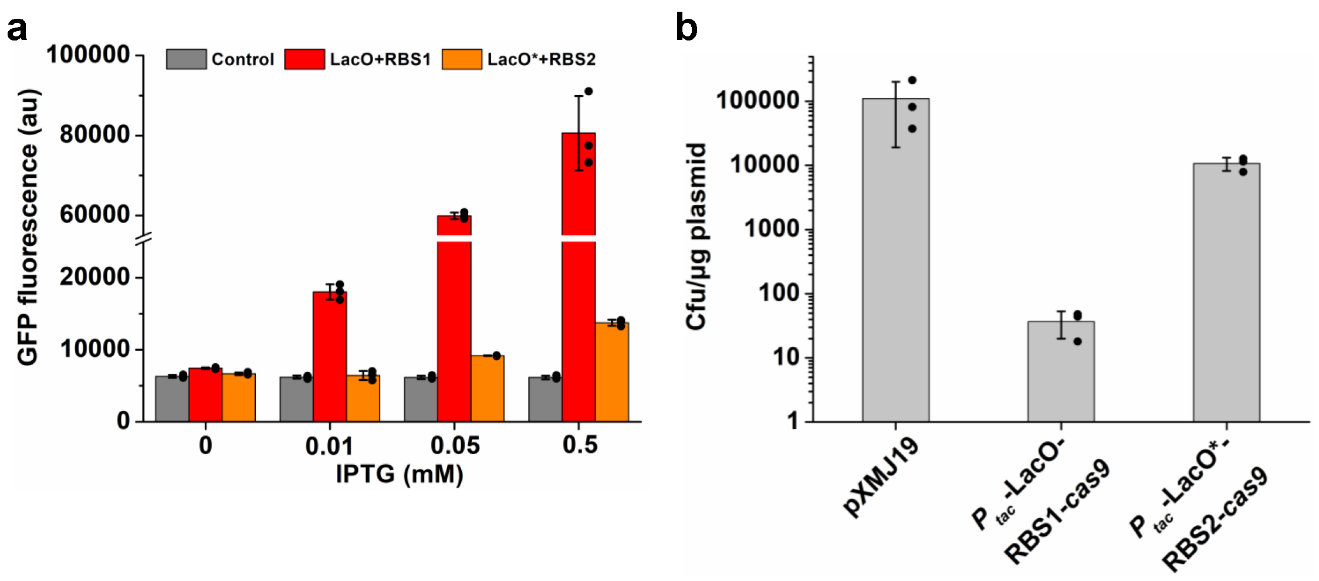


**Supplementary Fig. 2. Evaluation of the expression intensity, leakage, and cytotoxicity of the original and modified Cas9 expression systems.** **a**, *gfp* expression controlled by *P_tac_* promoter and LacO+RBS1 or LacO^*^+RBS2. LacO, wild-type *lac* operator; LacO^*^, a modified *lac* operator that binds LacI very tightly[^1^](#_ENREF_1); RBS1, a strong RBS AAAGGAGTTGAGA; RBS2, a weak RBS AAAGGCACCCGAT[^2^](#_ENREF_2). Plasmid pXMJ19 with an IPTG-inducible *P_tac_* promoter was used for *gfp* expression. *C. glutamicum* ATCC 13032 harboring a pXMJ19 empty plasmid was used as a control. Different concentrations of IPTG were added to induce *gfp* expression. Data are presented as mean values +/- SD (n = 3 independent experiments). **b**, Transformation efficiency of plasmids expressing *cas9* with *P_tac_* promoter and LacO+RBS1 or LacO^*^+RBS2. Plasmid pXMJ19 with an IPTG-inducible *P_tac_* promoter was used for *cas9* expression. pXMJ19 empty plasmid was used as a control. Data are presented as mean values +/- SD (n = 3 independent experiments). Source data underlying Supplementary Fig. 2 are provided as a Source Data file.


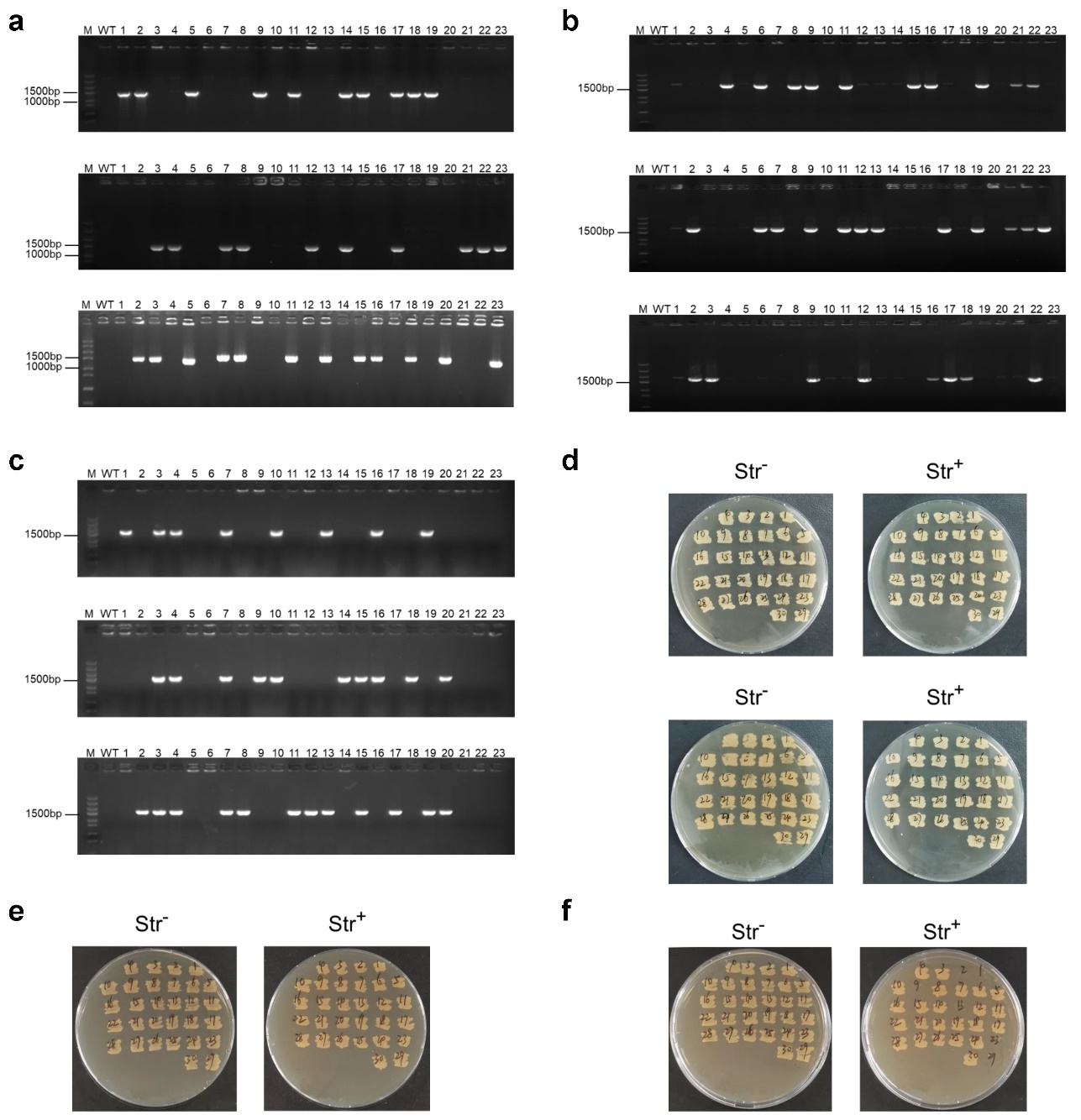


**Supplementary Fig. 3. PCR verification and streptomycin resistance phenotype test of CRISPR/Cas9-based gene deletion, insertion, and ssDNA recombineering in *C. glutamicum*.** **a**, Results for Fig. 1c, deletion of a 20 kb DNA fragment with 0.5 kb HR arms. The primers for colony PCR verification were designed at the upstream and downstream sites of the deleted fragment. No band was obtained for the wild-type control because the target 20 kb fragment was too large for PCR experiment conducted in this test. **b**, Results for Fig. 1c, deletion of a 219 kb DNA fragment with 1.0 kb HR arms. The primers for colony PCR verification were designed at the upstream and downstream sites of the deleted fragment. No band was obtained for the wild-type control because the target 219 kb fragment was too large for PCR experiment conducted in this test. **c**, Results for Fig. 1c, insertion of a 4 kb DNA fragment with 1.0 kb HR arms. The primers for colony PCR verification were designed at the inserted fragment. No band was obtained for the wild-type control because the wild-type strain does not contain the inserted artificial operon in is chromosome. For **a**, **b**, and **c**, three independent replicates were conducted and twenty-three colonies were randomly selected and verified by PCR for each test. **d**, Results for Fig. 1d, triple nucleotide changes with 90 nt ssDNA. The *rpsL*^K43R^ mutation produces streptomycin resistance phenotype. **e**, Results for Fig. 1d, single nucleotide change with 90 nt ssDNA. The *rpsL*^K43R^ mutation produces streptomycin resistance phenotype. **f**, Results for Fig. 1d, triple nucleotide changes with 60 nt ssDNA. The *rpsL*^K43R^ mutation produces streptomycin resistance phenotype.


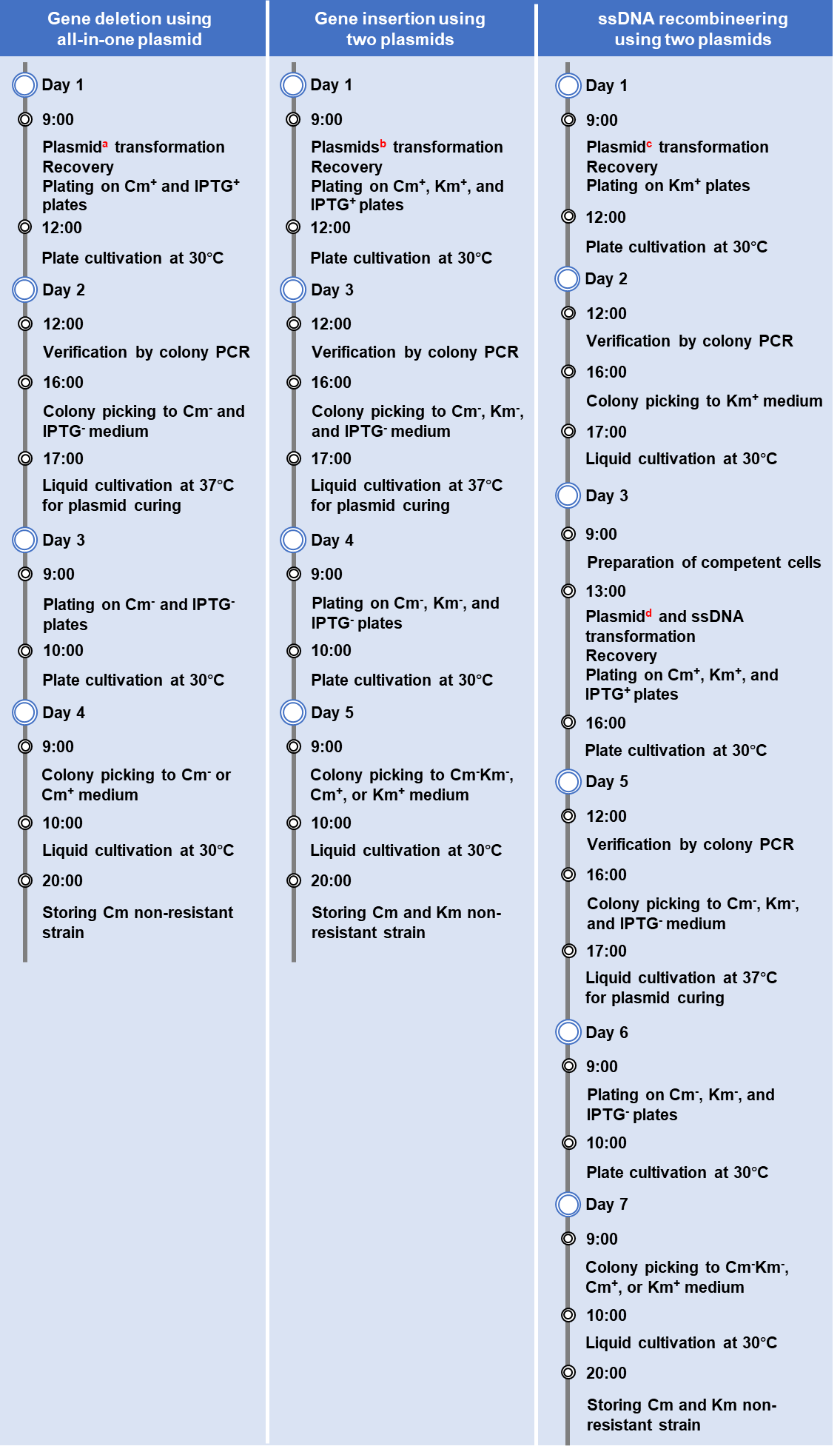


**Supplementary Fig. 4. Operation scheme of CRISPR/Cas9-assisted gene deletion, insertion, and ssDNA recombineering.** a, The all-in-one plasmid expressing Cas9 and gRNA and harboring homologous recombination arms is transformed. b, The plasmid expressing Cas9 and gRNA and another plasmid harboring homologous recombination arms and inserted gene fragments are co-transformed. c, The plasmid expressing RecT is transformed. d, The plasmid expressing Cas9 and gRNA is co-transformed with ssDNA. Km, kanamycin 25 μg/mL. Cm, chloramphenicol, 5 μg/mL. IPTG, 0.05 mM.


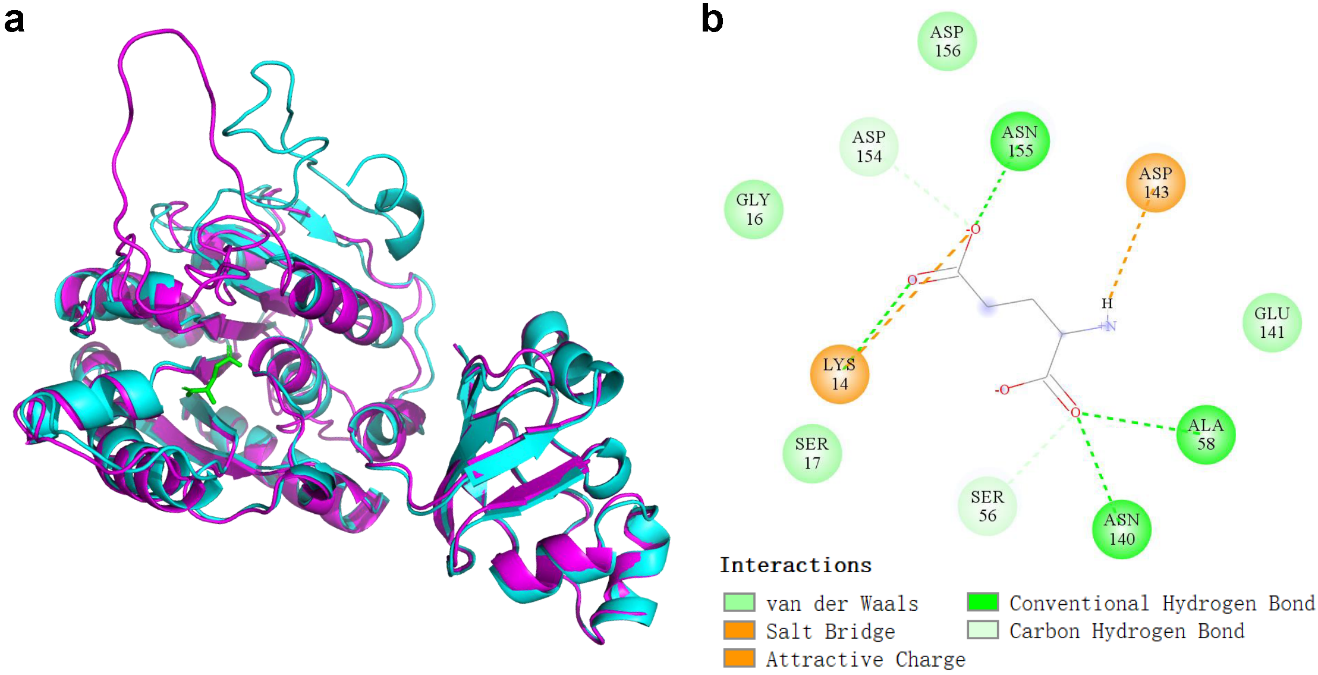


**Supplementary Fig. 5. Predicted structure and receptor-ligand interactions of** **γ-glutamyl kinase of *C. glutamicum* (CgProB).** **a**, Predicted structure of CgProB. The model structure was constructed with the crystal structure of γ-glutamyl kinase from *E. coli* (PDB ID: 2J5T)[^3^](#_ENREF_3) as a template (93% coverage and 38% sequence identity with CgProB) using Discovery Studio 2018 software (Accelrys, USA). Molecular docking with l-glutamate was performed using AutoDock Tools 1.5.6, and the optimal conformation was selected based on the method previously reported[^4^](#_ENREF_4). Cyan, the template (PDB ID: 2J5T); magenta, CgProB. The substrate l-glutamate is indicated in green. **b**, Analysis of the receptor-ligand interactions. Amino acid residuals with interaction with the substrate l-glutamate are shown. Receptor-ligand interaction analysis and figure rending were performed using Discovery Studio 2018 software (Accelrys, USA).


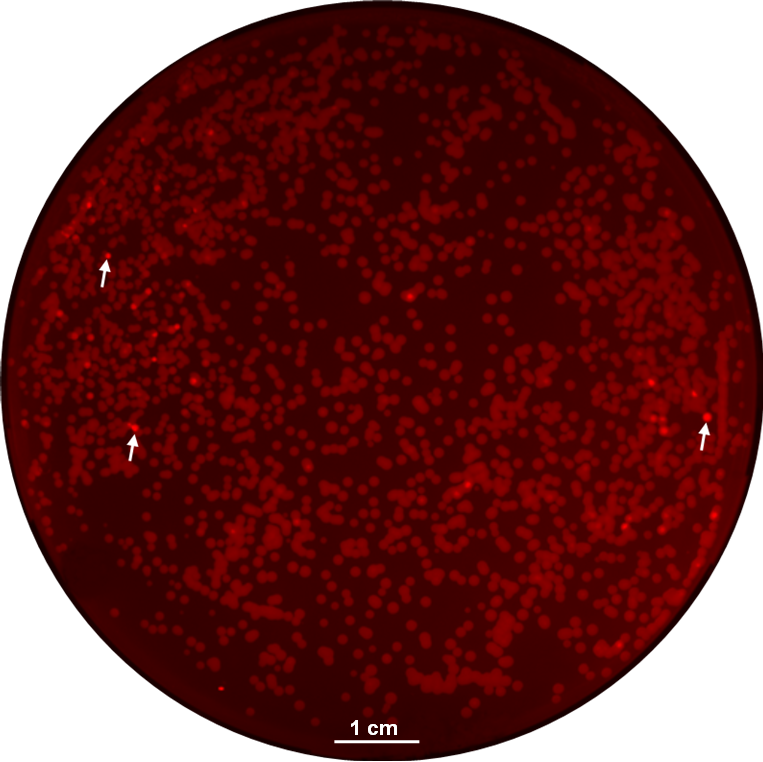


**Supplementary Fig. 6. Fluorescence imaging of transformants of promoter library on agar plates.** Colonies with enhanced RFP fluorescence are marked with white arrows. At least three independent replicates were conducted and the fluorescence image for one agar plate is shown.

**
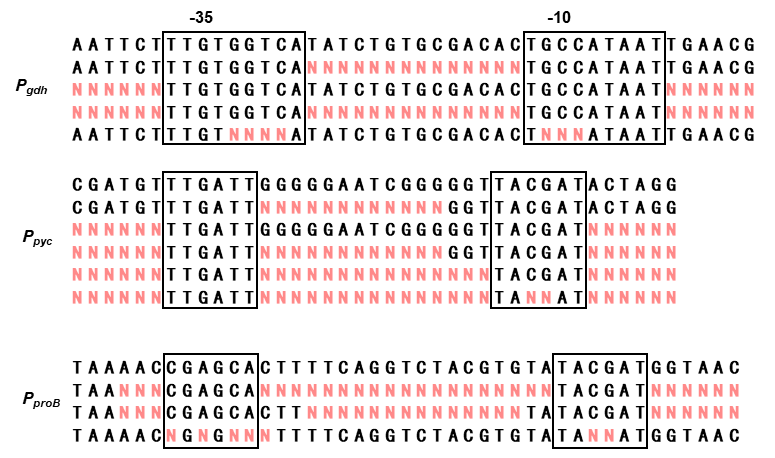
**

**Supplementary Fig. 7. Promoter libraries tested in this study.** The sequences of wild-type promoter and random mutation library are shown. The degenerate bases introduced during PCR primer synthesis are highlighted in red. The predicted -35 and -10 regions are highlighted in black boxes.


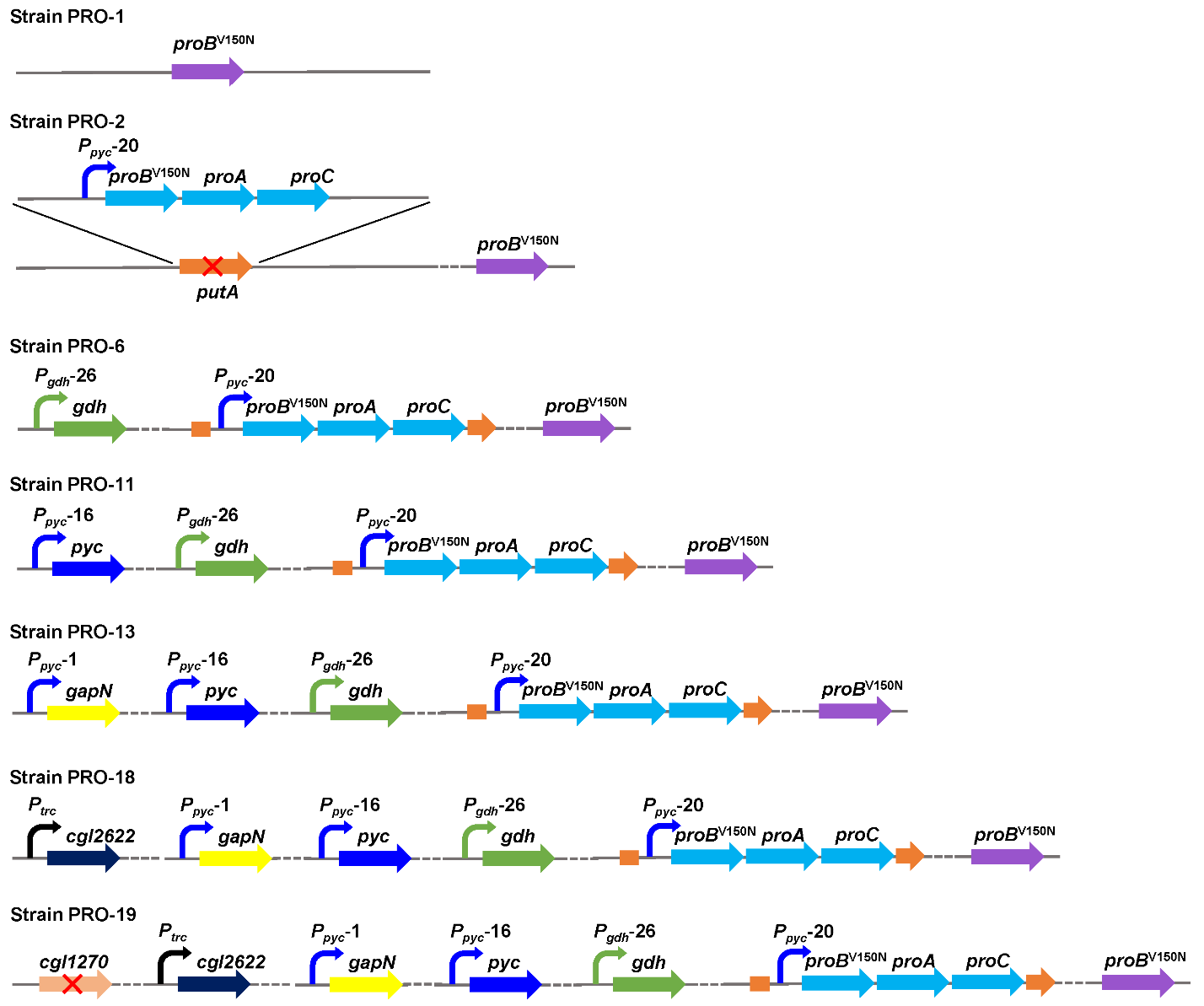


**Supplementary Fig. 8. Engineered strains for l-proline production.**





**Supplementary Fig. 9. Strength analysis of the five *P_pyc_* promoter variants using the *gapN*-*rfp* fusion gene as a reporter.** Cells withdrawn from different time points were used to detect their fluorescence outputs using a microplate reader (λ excitation = 560 nm, λ emission = 607 nm). Data are presented as mean values +/- SD (n = 3 independent experiments). Source data underlying Supplementary Fig. 9 are provided as a Source Data file.

**

**

**Supplementary Fig. 10. Intracellular l-proline concentration of l-proline producing strain PRO-13.** Strain PRO-13 was cultivated in shake flasks to obtain enough cells for the measurement of intracellular l-proline. Data are presented as mean values +/- SD (n = 3 independent experiments). Source data underlying Supplementary Fig. 10 are provided as a Source Data file.


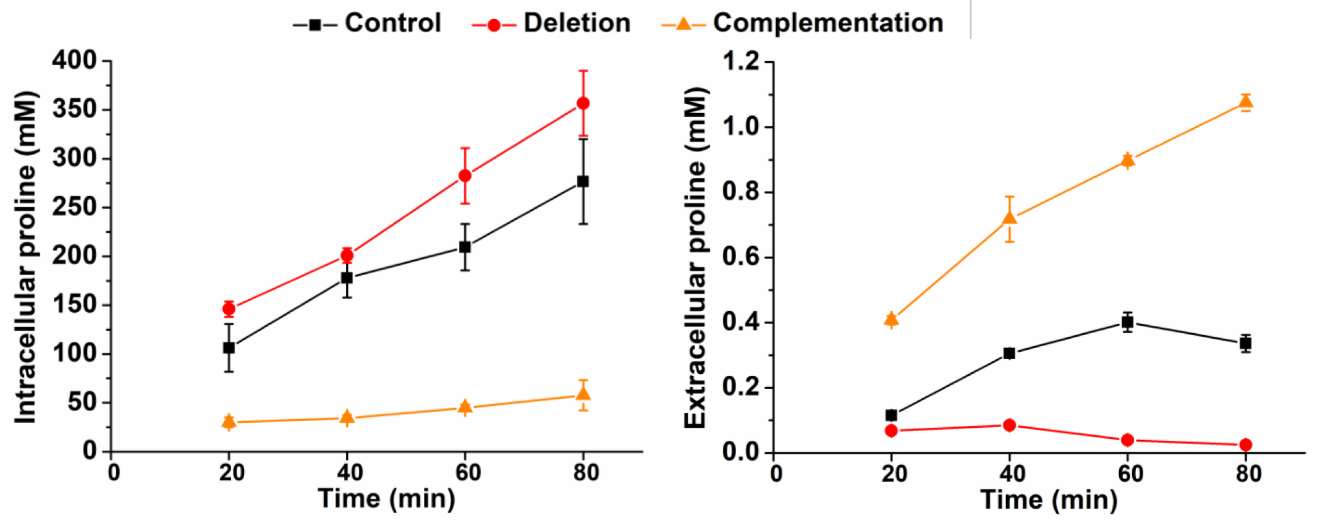


**Supplementary Fig. 11. Changes in intracellular and extracellular l-proline concentrations upon Thr-Pro peptide addition.** Control, *C. glutamicum* wild-type strain. Deletion, *C. glutamicum* Δ*cgl2622*. Complementation, *C. glutamicum* Δ*cgl2622* (pEC-8). pEC-8, pEC-XK99E carrying a *cgl2622* under the control of IPTG-inducible promoter *P_trc_*. Data are presented as mean values +/- SD (n = 3 independent experiments). Source data underlying Supplementary Fig. 11 are provided as a Source Data file.





**Supplementary Fig. 12. Effects of *cgl2622* overexpression on extracellular and intracellular accumulation of l-proline.** Strain PRO-18 was constructed by adding a second copy of *cgl2622* controlled by the derepressed *P_trc_* promoter in the chromosome of strain PRO-13. Strains were cultivated in shake flasks to obtain enough cells for the measurement of intracellular l-proline. Data are presented as mean values +/- SD (n = 3 independent experiments). **P*=0.0117, ***P*=0.0016, Student's two-tailed *t*-test. Source data underlying Supplementary Fig. 12 are provided as a Source Data file.


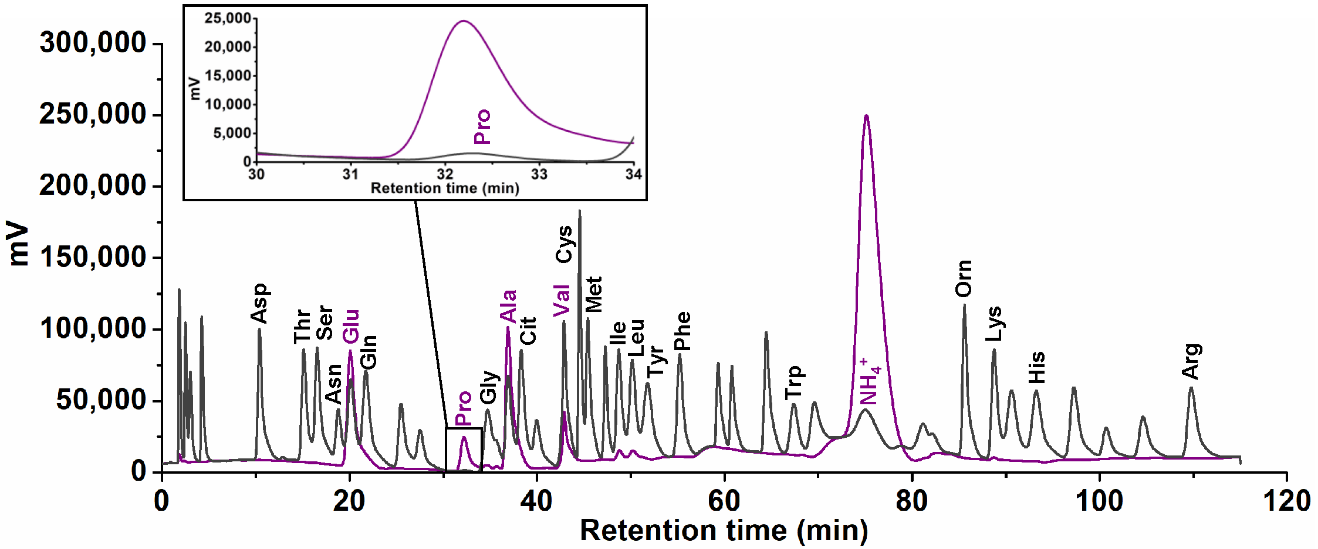


**Supplementary Fig. 13. Amino acid profile of the fermentation broth of strain PRO-19.** Amino acid profile was analyzed using the L-8900 Amino Acid Analyzer (Hitachi, Japan). The amino acids mixture standard solutions (Type AN-2 and Type B, FUJIFILM Wako Pure Chemical Corporation, Japan) were used as standards. Black, amino acid standard. Purple, 49-h fermentation broth of strain PRO-19. Twenty proteinogenic amino acids, l-ornithine (Orn), and l-citrulline (Cit) are marked on the profile. l-Proline has a low response factor using this analytical method and an amplified chromatogram is shown in the black box.

**Supplementary Table 1. Records of proteinogenic amino acid production by fed-batch fermentation of** **rationally engineered strains.**

| **Amino acid** | **Strain** | **Titer (g/L)** | **Yield (g/g)** | **Productivity**  **(g/L·h)** | **Ref.** |
| --- | --- | --- | --- | --- | --- |
| l-Lysine | *C. glutamicum* | 221.3 | 0.71 | 5.53 | [^5^](#_ENREF_5) |
| l-Glutamate^a^ | *C. glutamicum* | >150 | ～0.7 | NA^b^ | [^6^](#_ENREF_6) |
| l-Proline | *C. glutamicum* | 142.4 | 0.31 | 2.90 | This study |
|  | *C. glutamicum* | 120.18 | 0.20 | 1.581 | [^7^](#_ENREF_7) |
|  | *C. glutamicum* | 66.43 | 0.26 | 1.11 | [^8^](#_ENREF_8) |
| l-Threonine | *E. coli* | 116.62 | 0.486 | 2.43 | [^9^](#_ENREF_9) |
| l-Alanine | *E. coli* | 114.0 | 0.95 | 2.375 | [^10^](#_ENREF_10) |
| l-Arginine | *C. glutamicum* | 92.5 | 0.40 | 1.28 | [^11^](#_ENREF_11) |
| l-Valine | *E. coli* | 84 | 0.41 | 2.33 | [^12^](#_ENREF_12) |
| l-Glutamine | *C. glutamicum* | 73.5 | 0.368 | 1.11 | [^13^](#_ENREF_13) |
| l-Phenylalanine | *E. coli* | 72.9 | 0.26 | 1.40 | [^14^](#_ENREF_14) |
| l-Histidine | *E. coli* | 66.5 | 0.23 | 1.5 | [^15^](#_ENREF_15) |
| l-Tyrosine | *E. coli* | 55.54 | 0.25 | 1.38 | [^16^](#_ENREF_16) |
| l-Serine | *E. coli* | 50 | 0.36 | 1.06 | [^17^](#_ENREF_17) |
| l-Tryptophan | *E. coli* | 48.68 | 0.2187 | 1.28 | [^18^](#_ENREF_18) |
| l-Isoleucine | *C. glutamicum* | 32.1 | 0.181 | 0.45 | [^19^](#_ENREF_19) |
| l-Leucine | *C. glutamicum* | 24 | 0.219 | 0.564 | [^20^](#_ENREF_20) |

^a^The record for l-glutamate production was reported by the review of Ikeda and Takeno and the actual figures were not given[^6^](#_ENREF_6).

^b^NA, not available from the reference.

**Supplementary references**

1. Sadler, J.R., Sasmor, H. & Betz, J.L. A perfectly symmetric *lac* operator binds the *lac* repressor very tightly. *Proc. Natl. Acad. Sci. U. S. A.* **80,** 6785–6789 (1983).

2. Li, M. et al. Efficient multiplex gene repression by CRISPR-dCpf1 in *Corynebacterium glutamicum*. *Front. Bioeng. Biotechnol.* **8,** 357 (2020).

3. Marco-Marín, C. et al. A novel two-domain architecture within the amino acid kinase enzyme family revealed by the crystal structure of *Escherichia coli* glutamate 5-kinase. *J. Mol. Biol.* **367,** 1431–1446 (2007).

4. Trott, O. & Olson, A.J. AutoDock Vina: improving the speed and accuracy of docking with a new scoring function, efficient optimization, and multithreading. *J. Comput. Chem.* **31,** 455–461 (2010).

5. Xu, J.Z., Ruan, H.Z., Yu, H.B., Liu, L.M. & Zhang, W. Metabolic engineering of carbohydrate metabolism systems in *Corynebacterium glutamicum* for improving the efficiency of L-lysine production from mixed sugar. *Microb. Cell Fact.* **19,** 39 (2020).

6. Ikeda, M. & Takeno, S. Recent advances in amino acid production in *Corynebacterium glutamicum*: Biology and Biotechnology 10.1007/978-3-030-39267-3_7. (eds. M. Inui & K. Toyoda) 175–226 (Springer International Publishing, Cham; 2020).

7. Zhang, J. et al. *De novo* engineering of *Corynebacterium glutamicum* for L-proline production. *ACS Synth. Biol.* **9,** 1897–1906 (2020).

8. Zhang, Y. et al. A new genome-scale metabolic model of *Corynebacterium glutamicum* and its application. *Biotechnol. Biofuels* **10,** 169 (2017).

9. Zhao, L. et al. Expression regulation of multiple key genes to improve L-threonine in *Escherichia coli*. *Microb. Cell Fact.* **19,** 46 (2020).

10. Zhang, X., Jantama, K., Moore, J.C., Shanmugam, K.T. & Ingram, L.O. Production of L-alanine by metabolically engineered *Escherichia coli*. *Appl. Microbiol. Biotechnol.* **77,** 355–366 (2007).

11. Park, S.H. et al. Metabolic engineering of *Corynebacterium glutamicum* for L-arginine production. *Nat. Commun.* **5,** 4618 (2014).

12. Hao, Y. et al. High-yield production of L-valine in engineered *Escherichia coli* by a novel two-stage fermentation. *Metab. Eng.* **62,** 198–206 (2020).

13. Lv, Q. et al. Enhancing L-glutamine production in *Corynebacterium glutamicum* by rational metabolic engineering combined with a two-stage pH control strategy. *Bioresour. Technol.* **341,** 125799 (2021).

14. Liu, Y. et al. Genetic engineering of *Escherichia coli* to improve L-phenylalanine production. *BMC Biotechnol.* **18,** 5 (2018).

15. Wu, H. et al. Highly efficient production of L-histidine from glucose by metabolically engineered *Escherichia coli*. *ACS Synth. Biol.* **9,** 1813–1822 (2020).

16. Xu, S. et al. Construction of a heat-inducible *Escherichia coli* strain for efficient de novo biosynthesis of L-tyrosine. *Process Biochem.* **92,** 85–92 (2020).

17. Rennig, M. et al. Industrializing a bacterial strain for L-serine production through translation initiation optimization. *ACS Synth. Biol.* **8,** 2347–2358 (2019).

18. Wang, J. et al. Genetic engineering of *Escherichia coli* to enhance production of L-tryptophan. *Appl. Microbiol. Biotechnol.* **97,** 7587–7596 (2013).

19. Zhang, Y. et al. Metabolic engineering of *Corynebacterium glutamicum* WM001 to improve L-isoleucine production. *Biotechnol. Appl. Biochem.* **68,** 568–584 (2021).

20. Vogt, M. et al. Pushing product formation to its limit: metabolic engineering of *Corynebacterium glutamicum* for L-leucine overproduction. *Metab. Eng.* **22,** 40–52 (2014).
