# Supplementary material for: CRISPR-assisted rational flux-tuning and arrayed CRISPRi screening of an l-proline exporter for l-proline hyperproduction
Source: Nat Commun. 2022 Feb 16;13:891. doi: 10.1038/s41467-022-28501-7 (PMC8850433; doi:10.1038/s41467-022-28501-7)
Supplement: Supplementary file 2 — Description of Additional Supplementary Files [file 41467_2022_28501_MOESM2_ESM.pdf]

Description of Additional Supplementary Files

File Name: Supplementary Data 1

Description: ProB sequences used for sequence alignment.

File Name: Supplementary Data 2

Description: *In-silico* simulation of L-proline biosynthesis using *C. glutamicum* genome-scale metabolic model *i*CW773 (Maximum biomass, grey squares).

File Name: Supplementary Data 3

Description: *In-silico* simulation of L-proline biosynthesis using *C. glutamicum* genome-scale metabolic model *i*CW773 (Ppc pathway, purple squares).

File Name: Supplementary Data 4

Description: *In-silico* simulation of L-proline biosynthesis using *C. glutamicum* genome-scale metabolic model *i*CW773 (Pyc pathway, green squares).

File Name: Supplementary Data 5

Description: *In-silico* simulation of L-proline biosynthesis using *C. glutamicum* genome-scale metabolic model *i*CW773 (Pyc+GapN pathway, orange squares).

File Name: Supplementary Data 6

Description: Promoters used in this study.

File Name: Supplementary Data 7

Description: Predicted transporters in *C. glutamicum* and designed gRNAs.

File Name: Supplementary Data 8

Description: Strains and plasmids used in this study.

File Name: Supplementary Data 9

Description: Primers used in this study.

File Name: Supplementary Data 10

Description: ssDNAs used in this study.

File Name: Supplementary Data 11

Description: gRNAs used in this study.
