# Supplementary material for: CRISPR-assisted rational flux-tuning and arrayed CRISPRi screening of an l-proline exporter for l-proline hyperproduction
Source: Nat Commun. 2022 Feb 16;13:891. doi: 10.1038/s41467-022-28501-7 (PMC8850433; doi:10.1038/s41467-022-28501-7)
Supplement: Supplementary file 14 — Reporting Summary [file 41467_2022_28501_MOESM14_ESM.pdf]

nature portfolio

Corresponding author(s): Yu Wang

Last updated by author(s): Jan 16, 2022

Reporting Summary

Nature Portfolio wishes to improve the reproducibility of the work that we publish. This form provides structure for consistency and transparency in reporting. For further information on Nature Portfolio policies, see our Editorial Policies and the Editorial Policy Checklist.

Statistics

For all statistical analyses, confirm that the following items are present in the figure legend, table legend, main text, or Methods section.

n/a	Confirmed	
☐	☒	The exact sample size (*n*) for each experimental group/condition, given as a discrete number and unit of measurement
☐	☒	A statement on whether measurements were taken from distinct samples or whether the same sample was measured repeatedly
☐	☒	The statistical test(s) used AND whether they are one- or two-sided<br>*Only common tests should be described solely by name; describe more complex techniques in the Methods section.*
☒	☐	A description of all covariates tested
☒	☐	A description of any assumptions or corrections, such as tests of normality and adjustment for multiple comparisons
☐	☒	A full description of the statistical parameters including central tendency (e.g. means) or other basic estimates (e.g. regression coefficient) AND variation (e.g. standard deviation) or associated estimates of uncertainty (e.g. confidence intervals)
☐	☒	For null hypothesis testing, the test statistic (e.g. *F*, *t*, *r*) with confidence intervals, effect sizes, degrees of freedom and *P* value noted<br>*Give P values as exact values whenever suitable.*
☒	☐	For Bayesian analysis, information on the choice of priors and Markov chain Monte Carlo settings
☒	☐	For hierarchical and complex designs, identification of the appropriate level for tests and full reporting of outcomes
☒	☐	Estimates of effect sizes (e.g. Cohen's *d*, Pearson's *r*), indicating how they were calculated

Our web collection on statistics for biologists contains articles on many of the points above.

Software and code

Policy information about availability of computer code

Data collection	Red fluorescent protein outputs were detected using a microplate reader (SpectraMax M5, Molecular Devices, λ excitation = 560 nm, λ emission = 607 nm). Glucose in the medium was quantified using an SBA-40D biosensor analyzer (Institute of Biology of Shandong Province Academy of Sciences, Jinan, China). Extracellular L-proline concentrations of cultivation in deep-well plates and shake flasks were quantified according to the method based on the acid-ninhydrin reaction as described previously. Extracellular amino acids of cultivation in 5 L bioreactors were quantified using a HPLC method and an L-8900 Amino Acid Analyzer (Hitachi, Japan). Cell cultures were centrifuged at 13,000 × g for 5 min, and the supernatant was used for detection after appropriate dilution. The HPLC system consists of a Prominence UFLC (Shimadzu, Japan) equipped with a Zorbax Eclipse AAA column (4.6 mm × 150 mm, 5 μm, Agilent Technologies, USA) and a UV detector30. A gradient of 50 mM sodium acetate buffer at pH 6.4 with a gradient solution containing acetonitrile-water (50%, v/v) was used as the eluent. Amino acids were detected as their 2,4-dinitrofluorobenzene derivatives at 360 nm by following the precolumn derivation method. The L-8900 Amino Acid Analyzer (Hitachi, Japan) was used to analyze the amino acid profile of the fermentation broth of strain PRO-19 using the amino acids mixture standard solutions (Type AN-2 and Type B, FUJIFILM Wako Pure Chemical Corporation, Japan) as standards. Analysis was performed according to the manufacturer's instructions. Intracellular amino acids were extracted according to the procedure described previously and then quantified using the abovementioned HPLC method. The intracellular volume used to calculate the internal amino acid concentration was 1.7 μL/mg DCW. The model structure of CgProB was constructed with the crystal structure of γ-glutamyl kinase from E. coli (PDB ID: 2J5T) as a template using Discovery Studio 2018 software (Accelrys, USA). Molecular docking with L-glutamate was performed using AutoDock Tools 1.5.6, and the optimal conformation was selected based on the method previously reported. Receptor-ligand interaction analysis and figure rending were performed using Discovery Studio 2018 software (Accelrys, USA).

Data analysis	1. Error bars indicate standard deviations from three parallel experiments. All P values were generated from two-tailed t-tests using the Microsoft Excel 2016 (Microsoft Corporation).<br>2. The genome-scale metabolic model iCW773 was used to predict the optimal L-proline biosynthetic pathway in C. glutamicum by performing flux balance analysis. Simulations were performed using the COBRApy toolbox (v 0.22.1). Uptake rate of glucose was set as 10 mmol/gCDW·h.

For manuscripts utilizing custom algorithms or software that are central to the research but not yet described in published literature, software must be made available to editors and reviewers. We strongly encourage code deposition in a community repository (e.g. GitHub). See the Nature Portfolio guidelines for submitting code & software for further information.

Data

Policy information about availability of data

All manuscripts must include a data availability statement. This statement should provide the following information, where applicable:

- Accession codes, unique identifiers, or web links for publicly available datasets
- A description of any restrictions on data availability
- For clinical datasets or third party data, please ensure that the statement adheres to our policy

The data supporting the findings of this work are available within the paper and the Supplementary Information files. The crystal structure of γ-glutamyl kinase from E. coli (PDB ID: 2J5T, www.rcsb.org/structure/2J5T) was used as a template for modeling of CgProB. A reporting summary for this article is available as a Supplementary Information file. Source data are provided with this paper.

Field-specific reporting

Please select the one below that is the best fit for your research. If you are not sure, read the appropriate sections before making your selection.

[x] Life sciences [ ] Behavioural & social sciences [ ] Ecological, evolutionary & environmental sciences

For a reference copy of the document with all sections, see nature.com/documents/nr-reporting-summary-flat.pdf

Life sciences study design

All studies must disclose on these points even when the disclosure is negative.

Sample size	The sample size of experiments is determined at 3 (biological independent replicate) based on literature precedence for metabolic engineering experiments (Ref. https://doi.org/10.1038/s41467-020-16962-7; https://doi.org/10.1038/s41467-020-17223-3).
Data exclusions	No data were excluded from the analyses.
Replication	All experiments were repeated at least once. All attempts at replication were successful.
Randomization	E. coli and C. glutamicum strains used in this study were grown under identical conditions. For verification of bacterial transformants, colonies were randomly picked from the agar plates.
Blinding	Blinding was not performed in the study. Because 1) we need to know the performance of each engineered strain, 2) the samples of bacterial cultures were random sampling, and 3) the experiments did not involve any animals or human participants and the analysis was carried out entirely on bacteria.

Reporting for specific materials, systems and methods

We require information from authors about some types of materials, experimental systems and methods used in many studies. Here, indicate whether each material, system or method listed is relevant to your study. If you are not sure if a list item applies to your research, read the appropriate section before selecting a response.

Materials & experimental systems

n/a	Involved in the study	
[x]	[ ]	Antibodies
[x]	[ ]	Eukaryotic cell lines
[x]	[ ]	Palaeontology and archaeology
[x]	[ ]	Animals and other organisms
[x]	[ ]	Human research participants
[x]	[ ]	Clinical data
[x]	[ ]	Dual use research of concern

Methods

n/a	Involved in the study	
[x]	[ ]	ChIP-seq
[x]	[ ]	Flow cytometry
[x]	[ ]	MRI-based neuroimaging

March 2021
